# Anticancer evaluation and molecular docking of new pyridopyrazolo-triazine and pyridopyrazolo-triazole derivatives

**DOI:** 10.1038/s41598-023-29908-y

**Published:** 2023-02-16

**Authors:** Mohamed R. Elmorsy, Ehab Abdel-Latif, Hatem E. Gaffer, Samar E. Mahmoud, Ahmed A. Fadda

**Affiliations:** 1 Department of Chemistry, Faculty of Science, Mansoura University, Mansoura, 35516 Egypt; 2 Dyeing, Printing and Auxiliaries Department, National Research Centre, Cairo, 12622 Egypt

**Keywords:** Cancer, Chemistry

## Abstract

3-Amino-4,6-dimethylpyrazolopyridine was applied as a precursor for the synthesis of some new pyridopyrazolo-triazine and pyridopyrazolo-triazole derivatives through diazotization, followed by coupling with many 2-cyanoacetamide compounds, ethyl 3-(phenylamino)-3-thioxopropanoate, 3-oxo-*N*-phenylbutanethioamide, and *α*-bromo-ketone reagents [namely; 2-bromo-1-(4-fluorophenyl)ethan-1-one, 5-bromo-2-(bromoacetyl)thiophene, 3-(2-bromoacetyl)-2*H*-chromen-2-one and/or 3-chloroacetylacetone]. The prepared compounds were identified by spectroscopic analyses as IR, ^1^H NMR, and mass data. The anticancer activity of these pyrazolopyridine analogues was investigated in colon, hepatocellular, breast, and cervix carcinoma cell lines. The pyridopyrazolo-triazine compound **5a** substituted with a carboxylate group gave a distinguished value of IC_50_ = 3.89 µM against the MCF-7 cell line compared to doxorubicin as a reference drug. Also, the pyridopyrazolo-triazine compound **6a** substituted with the carbothioamide function gave good activity toward HCT-116 and MCF-7 cell lines with IC_50_ values of 12.58 and 11.71 µM, respectively. The discovered pyrazolopyridine derivatives were studied theoretically by molecular docking, and this study exhibited suitable binding between the active sides of pyrazolopyridine ligands and proteins (PDB ID: 5IVE). The pyridopyrazolo-triazine compound **6a** showed the highest free binding energy (− 7.8182 kcal/mol) when docked inside the active site of selected proteins.

## Introduction

**Figure 1:**
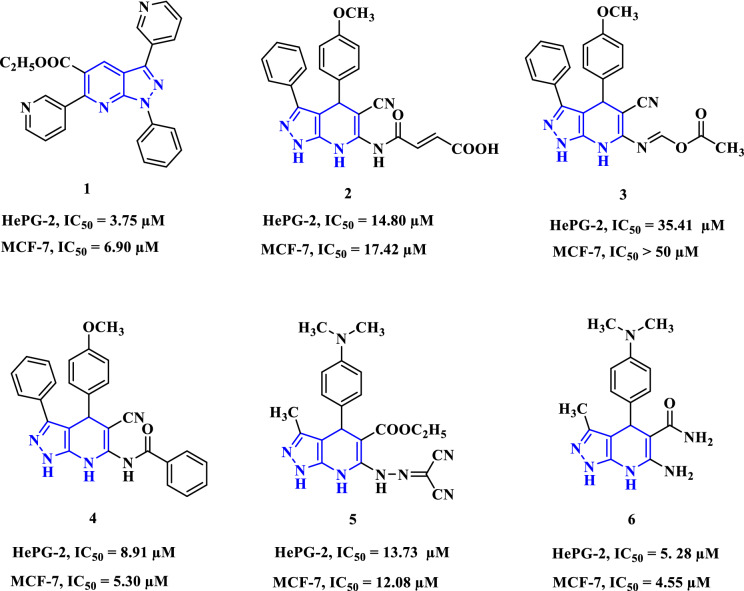
Anticancer activity of some known pyrazolopyridine derivatives.

Cancer is a fighting disease that can invade particular cells, damage their DNA, and then diffuse into other body cells, making it the second-leading cause of death in all undeveloped and developed countries^1–3^. Chemicals, UV radiation, the microbiome, and viruses are considered hazardous factors that contributed to the development of carcinoma. There are many types of tumors, such as lymphoma, leukemia, mamma, lung, liver, and colon cancer^4–6^. Most anticancer drugs can fiasco to treat infected cells due to several reasons; decreased water solubility of the drugs and diverse changes in cells that affect the ability of the drugs to kill cells, so researchers and scientists are studying how to overcome this^7^. Heterocyclic compounds containing nitrogen atoms are a unique class among the applied branches of organic chemistry and are important in all fields of physiological, biological, and medicinal science^8,9^. The compounds containing pyrazole have broad biological activities, such as antiviral^10,11^ and anti-inflammatory^12^. Fusing pyrazole with a pyridine nucleus gives different pyrazolopyridine systems that increase its pharmacological rank among other bioactive compounds^13–15^ (Fig. 1). One of the best-known pyrazole derivatives is amino pyrazolopyridine, which is an essential core structure in many drug substances and has important directions in bioactivities^16–18^ including antibacterial^19,20^, antifungal^21^, antitumor^22^, antiplatelet^23^, and antioxidant properties^24,25^. Based on these data, we aimed in this article to use the 3-amino-pyrazolopyridine derivative as a building block for tricyclic systems such as pyridopyrazolo-triazine and pyridopyrazolo-triazole analogues, which were synthesized by diazotization followed by diazocoupling with active methylene compounds, and esteem their antiproliferative ability against breast, colon, liver, and ovarian cancer cells. As well as theoretical evaluation by docking studies, which were done via MOE v. 2019.0102, and the physicochemical parameters by the Swiss ADME prediction website to predict the activity of newly synthesized compounds.

## Experimental

### Materials and methods

All reagents and solvents used in this synthesis were used without further purification. Melting points were obtained by the Gallenkamp apparatus. The IR spectra (KBr) were determined on a Thermo Scientific Nicolet iS10 FTIR spectrometer (Faculty of Science, Mansoura University). The ^1^H NMR spectra was recorded in DMSO-*d*_6_ using a JEOL’s NMR spectrometer (500 MHz) at Faculty of Science, Mansoura University. The mass analysis (molecular ion peaks) was measured using a Thermo Scientific GC/MS model ISQ and/or Agilent LC-MSD IQ Infinity II 1260 (Al-Azhar University, Egypt). Elemental analyses (C, H, and N) were performed with the Perkin-Elmer 2400 instrument (Microanalytically Unit, Cairo University). The anticancer activity evaluation was carried out at the bioassay-cell Culture Laboratory, National Research Centre, El-Tahrir Street, Dokki, Cairo, 12622, Egypt.

## Chemistry

### Synthesis of pyridopyrazolo-triazine compounds 3a-e

A suspension of 3-amino-4,6-dimethyl-1*H*-pyrazolo[3,4-*b*]pyridine (**1**) (0.81 g, 5 mmol) was dissolved in 1.50 mL conc. HCl and diazotized at 0–5 °C with an aqueous solution of NaNO_2_ (0.35 g, 5 mmol in 10 mL H_2_O). The freshly prepared diazonium chloride solution was added dropwise to cold solution of each 2-cyanoacetamide derivative (5 mmol) [namely; 2-cyano-*N*-phenylacetamide (0.80 g), *N*-(4-chlorophenyl)-2-cyanoacetamide (0.97 g), 2-cyano-*N*-(p-tolyl)acetamide (0.87 g), 2-cyano-*N*-(4-methoxyphenyl)acetamide (0.95 g), and *N*-(4-acetylphenyl)-2-cyanoacetamide (1.00 g) in 25 mL pyridine. When the addition is completed, the stirring was continued for 2 h and the solid that was obtained by filtration was dried and recrystallized from EtOH/AcOH mixture (1:2) to pick up the targeting pyridopyrazolo-triazine compounds **3a-e**.

#### 4-Amino-8,10-dimethyl-*N*-phenylpyrido[2′,3′:3,4]pyrazolo[5,1-*c*][1,2,4]triazine-3-carboxamide (3a)

Brown powder (48% yield); m.p. above 300 °C. IR (ῡ, cm^−1^): 3337, 3259, 3209 (N–H, NH_2_), 2923, 2853 (C–H, sp^3^), 1671 (C=O). ^1^H NMR (DMSO-*d*_6_): *δ* (ppm): 2.55 (s, 3H, CH_3_), 2.95 (s, 3H, CH_3_), 7.14 (t, *J* = 7.00 Hz, 1H, Ar–H), 7.19 (s, 1H, pyridine-H), 7.38 (t, *J* = 8.00 Hz, 2H, Ar–H), 7.92 (d, *J* = 8.00 Hz, 2H, Ar–H), 9.21 (s, 1H, N–H), 9.42 (s, 1H, N–H), 11.02 (s, 1H, N–H). Mass analysis (m/z, %): 333 (M^+^, 24.76), 324 (31.83), 321 (100.00), 318 (36.34), 307 (30.13), 306 (29.45), 298 (27.82), 295 (30.47), 284 (43.00), 261 (25.59), 248 (44.25), 132 (41.09). Analysis for C_17_H_15_N_7_O (333.13): Calculated: C, 61.25; H, 4.54; N, 29.41%. Found: C, 61.13; H, 4.51; N, 29.33%.

#### 4-Amino-*N*-(4-chlorophenyl)-8,10-dimethylpyrido[2′,3′:3,4]pyrazolo[5,1-*c*][1,2,4]triazine-3-carboxamide (3b)

Dark green powder (42% yield); m.p. above 300 °C. IR (ῡ, cm^−1^): 3396, 3351, 3204 (N–H, NH_2_), 2924, 2854 (C–H, sp^3^), 1662 (C=O). ^1^H NMR (DMSO-*d*_6_): *δ* (ppm): 2.65 (s, 3H, CH_3_), 2.93 (s, 3H, CH_3_), 7.16 (s, 1H, pyridine-H), 7.43 (d, *J* = 9.00 Hz, 2H, Ar–H), 7.97 (d, *J* = 9.00 Hz, 2H, Ar–H), 9.14 (s, 1H, N–H), 9.43 (s, 1H, N–H), 11.18 (s, 1H, N–H). Mass analysis (m/z, %): 367 (M^+^, 57.33), 360 (59.63), 342 (54.99), 283 (61.62), 269 (54.48), 228 (61.07), 223 (91.33), 199 (100.00), 174 (55.08), 157 (56.44), 152 (71.14), 137 (54.78), 94 (52.74), 91 (62.43), 84 (65.24), 82 (64.98), 80 (75.61), 73 (76.67). Analysis for C_17_H_14_ClN_7_O (367.09): Calculated: C, 55.52; H, 3.84; N, 26.66%. Found: C, 55.42; H, 3.81; N, 26.72%.

#### 4-Amino-8,10-dimethyl-*N*-(*p*-tolyl)pyrido[2′,3′:3,4]pyrazolo[5,1-*c*][1,2,4]triazine-3-carboxamide (3c)

Dark green powder (56% yield); m.p. above 300 °C. IR (ῡ, cm^−1^): 3398, 3336, 3259 (N–H, NH_2_), 2923, 2849 (C–H, sp^3^), 1671 (C=O). ^1^H NMR (DMSO-*d*_6_): *δ* (ppm): 2.28 (s, 3H, CH_3_), 2.68 (s, 3H, CH_3_), 2.96 (s, 3H, CH_3_ ), 7.17 (d, *J* = 8.50 Hz, 2H, Ar–H), 7.21 (s, 1H, pyridine-H), 7.79 (d, *J* = 8.50 Hz, 2H, Ar–H), 9.26 (s, 1H, N–H), 9.40 (s, 1H, N–H), 10.94 (s, 1H, N–H). Mass analysis (m/z, %): 347 (M^+^, 25.80), 335 (27.72), 318 (100.00), 221 (24.52), 177 (23.85), 132 (27.75), 87 (33.17), 83 (37.66), 58 (28.49), 52 (25.65), 43 (29.32). Analysis for C_18_H_17_N_7_O (347.15): Calculated: C, 62.24; H, 4.93; N, 28.23%. Found: C, 62.38; H, 4.87; N, 28.32%.

#### 4-Amino-*N*-(4-methoxyphenyl)-8,10-dimethylpyrido[2′,3′:3,4]pyrazolo[5,1-*c*][1,2,4]triazine-3-carboxamide (3d)

Dark green powder (54% yield); m.p. above 300 °C. IR (ῡ, cm^−1^): 3352, 3267 3194 (N–H, NH_2_), 2923, 2853 (C–H, sp^3^), 1668 (C = O). ^1^H NMR (DMSO-*d*_6_): *δ* (ppm): 2.66 (s, 3H, CH_3_), 2.95 (s, 3H, CH_3_), 3.75 (s, 3H, OCH_3_), 6.95 (d, *J* = 9.00 Hz, 2H, Ar–H), 7.18 (s, 1H, pyridine-H), 7.82 (d, *J* = 9.00 Hz, 2H, Ar–H), 9.22 (s, 1H, N–H), 9.38 (s, 1H, N–H), 10.95 (s, 1H, N–H). Mass analysis (m/z, %): 363 (M^+^, 56.51), 347 (56.31), 335 (61.83), 333 (68.60), 331 (85.63), 302 (60.14), 294 (78.10), 284 (81.75), 281 (63.40), 265 (64.97), 259 (63.63), 213 (85.50), 194 (64.61), 189 (65.10), 150 (63.08), 117 (100.00), 112 (61.24). Analysis for C_18_H_17_N_7_O_2_ (363.14): Calculated: C, 59.50; H, 4.72; N, 26.98%. Found: C, 59.60; H, 4.74; N, 26.93%.

#### *N*-(4-Acetylphenyl)-4-amino-8,10-dimethylpyrido[2′,3′:3,4]pyrazolo[5,1-*c*][1,2,4]triazine-3-carboxamide (3e)

Yellow powder (58% yield); m.p. above 300 °C. IR (ῡ, cm^−1^): 3371, 3266, 3201 (N–H, NH_2_), 2921, 2838 (C–H, sp^3^), 1682 (C=O), and 1641 (C=O). ^1^H NMR (DMSO-*d*_6_): *δ* (ppm): 2.55 (s, 3H, COCH_3_), 2.65 (s, 3H, CH_3_), 2.93 (s, 3H, CH_3_), 7.16 (s, 1H, pyridine-H), 7.98 (d, *J* = 9.00 Hz, 2H, Ar–H), 8.09 (d, *J* = 9.00 Hz, 2H, Ar–H), 9.14 (s, 1H, N–H), 9.45 (s, 1H, N–H), 11.29 (s, 1H, N–H). Mass analysis (m/z, %): 375 (M^+^, 42.72), 292 (42.95), 284 (43.36), 281 (47.71), 266 (43.31), 262 (43.76), 256 (52.97), 252 (68.53), 249 (44.67), 216 (55.10), 203 (44.90), 182 (52.38), 148 (52.43), 128 (52.56), 81 (45.85), 71 (100.00), 64 (52.93). Analysis for C_19_H_17_N_7_O_2_ (375.14): Calculated: C, 60.79; H, 4.56; N, 26.12%. Found: C, 60.66; H, 4.51; N, 26.21%.

### Synthesis of pyridopyrazolo-triazine-3-carboxylate compounds 5a, 6a, and 7

The cold solution of sodium nitrite (0.35 g in 10 mL of water) was added drop by drop to a cold solution of 3-amino-4,6-dimethyl-1*H*-pyrazolo[3,4-*b*]pyridine (**1**) (0.81 g, 5 mmol) and 1.5 mL of conc. HCl. Then the solution of ethyl 3-(phenylamino)-3-thioxopropanoate **(4a)** (1.1 mL, 5 mmol), 3-oxo-*N*-phenylbutanethioamide **(4b)** (0.96 mL, 5 mmol), and ethyl 4-chloro-3-oxobutanoate **(4c)** (0.68 mL, 5 mmol) in 25 mL ethanol and sodium acetate (1.50 g) was treated with the prepared diazonium solution at 0–5 °C. The reaction mixture was stirred for 2 h. The solid product obtained was filtered off, washed with water, and recrystallized from ethanol to give pyridopyrazolo-triazine derivatives **5a**, **6a**, and** 7**, respectively.

#### Ethyl 4-mercapto-8,10-dimethylpyrido[2′,3′:3,4]pyrazolo[5,1-*c*][1,2,4]triazine-3-carboxylate (5a)

Orange powder (61% yield); m.p. = 230–232 °C. IR (ῡ, cm^−1^): 2979, 2925 (C–H, sp^3^), 1734 (C=O). ^1^H NMR (DMSO-*d*_6_): *δ* (ppm): 1.49 (t, *J* = 7.00 Hz, 3H, CH_3_), 2.85 (s, 6H, 2CH_3_), 4.57 (q, *J* = 7.00 Hz, 2H, CH_2_), 6.85 (s, 1H, pyridine-H), 14.82 (s, 1H, S–H). Mass analysis (m/z, %): 304 (M^+^ + 1, 100.00). Analysis for C_13_H_13_N_5_O_2_S (303.08): Calculated: C, 51.47; H, 4.32; N, 23.09%. Found: C, 51.36; H, 4.28; N, 23.16%.

#### 4,810-Trimethyl-*N*-phenylpyrido[2′,3′:3,4]pyrazolo[5,1-*c*][1,2,4]triazine-3-carbothioamide (6a)

Greenish yellow powder (67% yield); m.p. 240–242 °C. IR (ῡ, cm^−1^): 3232 (N–H), 2973, 2927 (C–H, sp^3^). ^1^H NMR (DMSO-*d*_6_): *δ* (ppm): 2.71 (s, 3H, CH_3_), 3.01 (s, 6H, 2CH_3_), 7.34–7.37 (t, *J* = 9.00 Hz, 2H, Ar–H and pyridine-H), 7.52 (t, *J* = 8.00 Hz, 2H, Ar–H), 8.04 (d, *J* = 7.50 Hz, 2H, Ar–H), 12.59 (s, 1H, N–H). Mass analysis (m/z, %): 348 (M^+^, 41.57), 339 (40.55), 328 (25.85), 250 (16.63), 241 (20.44), 189 (18.87), 181 (27.29), 170 (27.54), 169 (19.86), 83 (66.55), 71 (49.65), 68 (100.00), 60 (73.40). Analysis for C_18_H_16_N_6_S (348.12): Calculated: C, 62.05; H, 4.63; N, 24.12%. Found: C, 62.18; H, 4.59; N, 24.03%.

#### Ethyl 4-(chloromethyl)-8,10-dimethylpyrido[2',3':3,4]pyrazolo[5,1-*c*][1,2,4]triazine-3-carboxylate (7)

Orange powder (54% yield); m.p. 160–162 °C. IR (ῡ, cm^−1^): 2974, 2925 (C–H, sp^3^), 1736 (C=O). ^1^H NMR (DMSO-*d*_6_): *δ* (ppm): 1.55 (t, *J* = 7.00 Hz, 3H, CH_3_), 2.83 (s, 3H, CH_3_), 3.10 (s, 3H, CH_3_), 4.63 (q, *J* = 7.00 Hz, 2H, CH_2_), 5.77 (s, 2H, CH_2_), 7.29 (s, 1H, pyridine-H). ^13^C NMR (*δ*/ppm): 14.19, 19.35, 25.91, 34.26, 63.30, 105.65, 122.05, 133.83, 135.86, 145.47, 145.66, 161.80, 163.23, 166.71. Mass analysis (m/z, %): 319 (M^+^, 10.96), 267 (43.74), 225 (66.05), 198 (46.40), 167 (54.39), 159 (56.84), 125 (51.46), 101 (80.29), 88 (78.17), 84 (64.38), 75 (79.25), 63 (98.90), 59 (100.00). Analysis for C_14_H_14_ClN_5_O_2_ (319.08): Calculated: C, 52.59; H, 4.41; N, 21.90%. Found: C, 52.43; H, 4.38; N, 21.80%.

### Synthesis of pyridopyrazolo-triazole compounds 9a, 9b, 10 and 11

A solution of sodium nitrite (0.35 g in 10 mL water) was added drop by drop to a suspension of 3-amino-4,6-dimethyl-1*H*-pyrazolo[3,4-*b*]pyridine (**1**) (0.81 g, 5 mmol) in 1.5 mL of conc. HCl at 0–5 °C. Then the freshly obtained diazonium solution was added drop by drop to a suspension of each α-bromo-ketone reagents **8a-d** (5 mmol) [namely; 2-bromo-1-(4-fluorophenyl)ethan-1-one (1 g), 2-bromo-1-(4-chlorophenyl)ethan-1-one (1.16 g), 2-bromo-1-(5-bromothiophen-2-yl)ethan-1-one (1.41 g), and/or 3-(2-bromoacetyl)-2*H*-chromen-2-one (1.33 g)] in 25 mL pyridine. The stirring time was continued at 0–5 °C for 2 h and the solid that yielded was filtered and purified from EtOH/DMF mixture (1:1) to afford pyridopyrazolo-triazole derivatives **9a**, **9b**, **10** and **11**, respectively.

#### (7,9-Dimethyl-1*H*-[1,2,4]triazolo[4',3':1,5]pyrazolo[3,4-*b*]pyridin-3-yl)(4-fluorophenyl)methanone (9a)

Red powder (60% yield); m.p. above 300 °C. IR (ῡ, cm^−1^): 3159 (N–H), 2923, 2851 (C–H, sp^3^), 1660 (C=O). ^1^H NMR (DMSO-*d*_6_): *δ* (ppm): 2.61 (s, 3H, CH_3_), 2.86 (s, 3H, CH_3_), 6.89 (s, 1H, pyridine-H), 7.44 (t, *J* = 9.00 Hz, 2H, Ar–H), 8.56 (t, *J* = 9.00 Hz, 2H, Ar–H), 14.51 (s, 1H, N–H). Mass analysis (m/z, %): 309 (M^+^, 11.33), 285 (30.24), 284 (24.83), 267 (25.59), 254 (24.06), 232 (28.74), 230 (30.67), 228 (100.00), 201 (34.56), 165 (53.45), 119 (64.94), 118 (39.83), 116 (29.58), 99 (87.66). Analysis for C_16_H_12_FN_5_O (309.10): Calculated: C, 62.13; H, 3.91; N, 22.64%. Found: C, 62.28; H, 3.94; N, 22.71%.

#### (4-Chlorophenyl)(7,9-dimethyl-1*H*-[1,2,4]triazolo[4′,3′:1,5]pyrazolo[3,4-*b*]pyridin-3-yl)methanone (9b)

Yellow powder (69% yield); m.p. above 300 °C. IR (ῡ, cm^−1^): 3185 (N–H), 2925, 2814 (C–H, sp^3^), 1631 (C=O). ^1^H NMR (DMSO-*d*_6_): *δ* (ppm): 2.60 (s, 3H, CH_3_), 2.84 (s, 3H, CH_3_), 6.85 (s, 1H, pyridine-H), 7.67 (d, *J* = 8.50 Hz, 2H, Ar–H), 8.47 (d, *J* = 8.50 Hz, 2H, Ar–H), 14.48 (s, 1H, N–H). Mass analysis (m/z, %): 325 (M^+^, 37.97), 313(44.51), 305 (52.14), 289 (100.00), 275 (76.07), 243 (44.46), 213 (88.63), 175 (57.49), 174 (46.08), 172 (43.49), 146 (57.32), 100 (47.94), 98 (44.34), 74 (48.07). Analysis for C_16_H_12_ClN_5_O (325.07): Calculated: C, 58.99; H, 3.71; N, 21.50%. Found: C, 58.89; H, 3.69; N, 21.55%.

#### (5-Bromothiophen-2-yl)(7,9-dimethyl-1*H*-[1,2,4]triazolo[4′,3′:1,5]pyrazolo[3,4-*b*]pyridin-3-yl)methanone (10)

Pale brown powder (54% yield); m.p. above 300 °C. IR (ῡ, cm^−1^): 3349 (N–H), 2922, 2849 (C–H, sp^3^), 1631 (C=O). ^1^H NMR (DMSO-*d*_6_): *δ* (ppm): 2.61 (s, 3H, CH_3_), 2.85 (s, 3H, CH_3_), 6.89 (s, 1H, pyridine-H), 7.47 (d, *J* = 4.00 Hz, 1H, thiophene-H), 8.33 (d, *J* = 4.00 Hz, 1H, thiophene-H), 14.56 (s, 1H, N–H). Mass analysis (m/z, %): 376 (M^+^, 17.92), 369 (69.63), 352 (91.00), 316 (47.28), 304 (65.20), 288 (42.20), 280 (67.34), 265 (52.43), 256 (62.48), 245 (46.84), 241 (55.62), 239 (42.45), 231 (53.37), 168 (61.39), 136 (57.04), 99 (50.80), 97 (63.82), 85 (60.16), 63 (100.00). Analysis for C_14_H_10_BrN_5_OS (374.98): Calculated: C, 44.69; H, 2.68; N, 18.61%. Found: C, 44.79; H, 2.69; N, 18.64%.

#### 3-(7,9-Dimethyl-1*H*-[1,2,4]triazolo[4′,3′:1,5]pyrazolo[3,4-*b*]pyridine-3-carbonyl)-2*H*-chromen-2-one (11)

Brown powder (60% yield); m.p. above 300 °C. IR (ῡ, cm^−1^): 3228 (N–H), 2922, 2846 (C–H, sp^3^), 1724 (C=O), 1610 (C=O). ^1^H NMR (DMSO-*d*_6_): *δ* (ppm): 2.35 (s, 6H, 2CH_3_), 6.78 (s, 1H, pyridine-H), 7.40 (t, *J* = 9.50 Hz, 2H, Ar–H), 7.63–6.69 (m, 1H, Ar–H), 7.88 (s, 1H, Ar–H), 8.64 (s, 1H, Ar–H). Mass analysis (m/z, %): 359 (M^+^, 36.20), 356 (54.35), 304 (67.94), 303 (56.46), 294 (45.50), 292 (58.05), 282 (43.83), 272 (54.12), 170 (48.48), 161 (100.00), 108 (57.07), 104 (62.27), 100 (44.12). Analysis for C_19_H_13_N_5_O_3_ (359.10): Calculated: C, 63.51; H, 3.65; N, 19.49%. Found: C, 63.64; H, 3.62; N, 19.55%.

#### Synthesis of 1-(7,9-Dimethyl-1*H*-[1,2,4]triazolo[4′,3′:1,5]pyrazolo[3,4-*b*]pyridin-3-yl)ethan-1-one (12)

A solution of sodium nitrite (0.35 g in 10 mL water) was added drop by drop to a suspension of 3-amino-4,6-dimethyl-1*H*-pyrazolo[3,4-*b*]pyridine (**1**) (0.81 g, 5 mmol) in 1.5 mL of conc. HCl at 0–5 °C. Then the freshly obtained diazonium solution was added drop by drop to a suspension of each 3-chloroacetylacetone (0.56 mL, 5 mmol) in 30 mL ethanol and 1.50 f. sodium acetate. The stirring time was continued at 0–5 °C for 2 h and the solid that yielded was filtered and recrystallized from ethanol to afford pyridopyrazolo-triazole derivative **12**. Yellow powder (73% yield); m.p. = 180–182 °C. IR (ῡ, cm^−1^): 3290 (N–H), 2984, 2927 (C–H, sp^3^), 1679 (C=O). ^1^H NMR (DMSO-*d*_6_): *δ* (ppm): 2.19 (s, 3H, CH_3_), 2.56 (s, 3H, CH_3_), 2.69 (s, 3H, CH_3_), 7.48 (s, 1H, pyridine-H), 14.33 (s, 1H, N–H). Mass analysis (m/z, %): 229 (M^+^, 100.00). Analysis for C_11_H_11_N_5_O (229.10): Calculated: C, 57.63; H, 4.84; N, 30.55%. Found: C, 57.51; H, 4.87; N, 30.64%.

## Biological evaluation

### Cytotoxicity MTT assay

The cytotoxicity of all novel pyridopyrazolo-triazine and pyridopyrazolo-triazole derivatives was assessed using the MTT assay on four different types of cell lines; colorectal carcinoma (HCT-116), hepatocellular carcinoma (HEPG-2), breast cancer (MCF-7) and cervix cancer (Hela). We obtained the cell lines from ATCC via a holding company for vaccines and biological products (Viscera). With the MTT assay, the above-mentioned cell lines were used to see if test compounds stopped the growth of cells^26,27^.The cell lines were introduced into 96-well plates with a concentration of 1 × 10^4^ cells per well in 100 μL of complete medium. After 24 h, the reference drug (Doxorubicin) and various concentrations of the samples (100, 50, 25, 12.5, 6.25, 3.125, and 1.56 μM) were prepared for maintenance media and added. These plates were incubated for 24 h, 5% CO_2_, at 37 °C. The MTT solution (20 μL, 5 mg/mL) was added to the plates and incubated for 4 h at 37 °C. 100 μL of DMSO was added to each well to solubilize the formazan. The absorbance was measured at 540 nm by a plate reader. The percentage of viability was calculated as (A570 of treated samples/A570 of untreated samples) × 100.

### Molecular docking study

The docking study is useful because of its capability to foretell the preferred orientation of small ligands to the suitable binding site of the target receptor. A molecular docking study was performed through the crystal structure of the protein^28^. The crystal structure (PDB ID: 5IVE) was deduced from the Protein Data Bank file (PDB) and performed by utilizing MOE v. 2019.0102^29^. The protein preparation was carried out as follows: Water and hetero residues were ignored, and missing hydrogen atoms were added to the enzyme skeleton before the docking process to achieve the correct ionization states for the amino acids. The active site of the protein was also detected within 10 Å of the ligand. Finally, binding energies of ligand-receptor interactions were recorded.

## Results and discussion

### Chemistry

**Figure 2:**
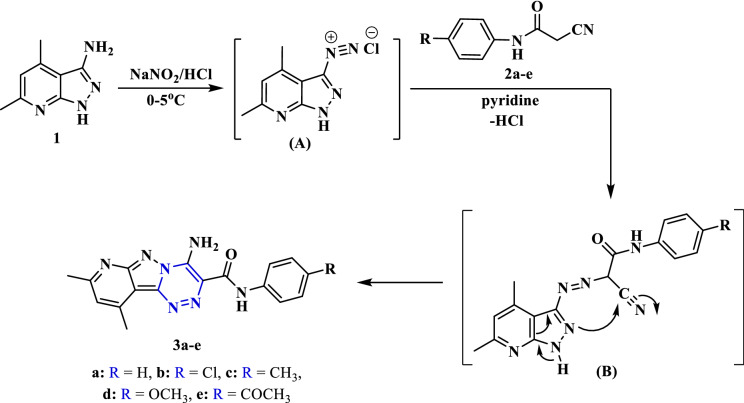
Synthesis of pyridopyrazolo-triazine derivatives **3a-e**.

3-Amino-4,6-dimethyl-1*H*-pyrazolo[3,4-*b*]pyridine (**1**)^30^ was diazotized using nitrous acid (NaNO_2_/HCl) at 0–5 °C. The diazotized aromatic amine **(A)** was coupled with different 2-cyanoacetamide derivatives **2a-e**^31^ to produce the arylazo-cyanoacetamide intermediate (**B**) that cyclized via addition of the cyclic NH into the nitrile group to form a tricyclic system, pyridopyrazolo-triazine derivatives **3a-e** (Fig. 2). This diazo-coupling reaction was carried out in pyridine. The spectroscopic analyses (IR, ^1^H NMR, and MS) have been utilized to confirm the structures of the target pyridopyrazolo-triazine derivatives **3a-e**. The IR spectrum of compound **3c** displayed absorption bands at 3398, 3336, and 3259 cm^−1^ for the N–H and –NH_2_ groups, in addition to the absorption at 1671 cm^−1^ for the carbonyl group. Its ^1^H NMR spectrum of compound **3c** showed three singlet signals at *δ* 2.28, 2.68, and 2.96 ppm for three methyl groups. The proton of the pyridine ring was observed as singlet at *δ* 7.21 ppm, while the aromatic protons were resonated as two doublet signals at *δ* 7.17 and 7.79 ppm. Further, the three singlet signals at *δ* 9.26, 9.40, and 10.94 ppm were attributed to the protons of three NH groups. Moreover, the mass analysis of compound **3c** identified the molecular ion peak at *m/z* = 347 (25.80%), which corresponds to a molecular formula (C_18_H_17_N_7_O).

**Figure 3:**
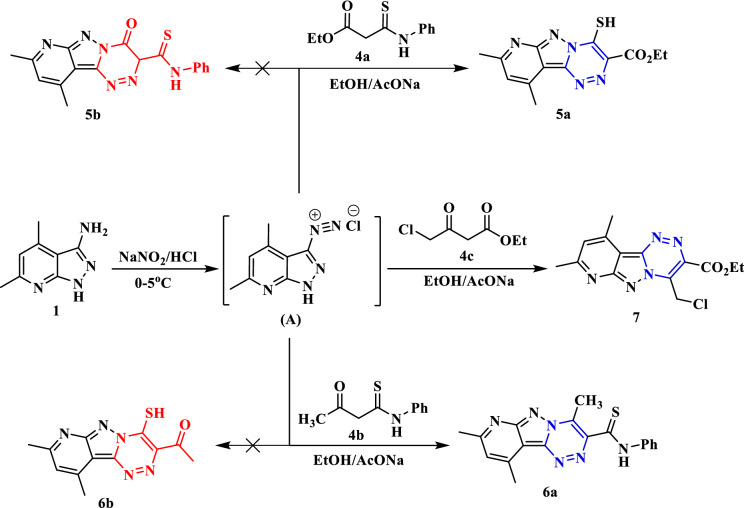
Synthesis of pyridopyrazolo-triazine derivatives **5a**, **6a**, and **7**.

The electrophilic coupling reaction of ethyl 3-(phenylamino)-3-thioxopropanoate (**4a**) with the diazonium salt derived from 4,6-dimethyl-1*H*-pyrazolo[3,4-*b*]pyridin-3-amine (**1**) was carried out in ethanol and sodium acetate at 0-5ºC to furnish the conforming ethyl4-mercapto-8,10-dimethylpyrido[2′,3′:3,4]pyrazolo[5,1-*c*][1,2,4]triazine-3-carboxylate (**5a**). The cyclization proceeded by nucleophilic addition of the N–H on the thioamide function (-CSNHPh) followed by loss of the aniline molecule to produce the pyridopyrazolo-triazine compound **5a**. The expected cyclization, through addition of the N–H group to the ester function (–COOEt) followed by loss of the ethanol molecule to form compound **5b**, did not match the spectroscopic analyses. The structure of the pyridopyrazolo-triazine **5a** was confirmed by the IR spectrum, which displayed characterized absorption at 1734 cm^-1^ for the carbonyl group (C=O). The ^1^H NMR spectrum indicated the signals of the ethoxy group (ester function) as a triplet at *δ* 1.49 ppm for the protons of the methyl group and a quartet for the protons of the methylene group at *δ* 4.57 ppm. The singlet that was observed at 14.82 ppm is attributed to the proton of the thiol (–SH) group. Further, the coupler 3-oxo-*N*-phenylbutanethioamide (**4b**) was reacted with the diazonium salt (**A**) in the presence of ethanol and sodium acetate. The cyclization occurred through the nucleophilic addition of the N–H on the acetyl group followed by the loss of a water molecule to yield the corresponding pyridopyrazolo-triazine **6a** instead of the alternative cyclization that produced the pyridopyrazolo-triazine **6b**. The skeleton of pyridopyrazolo-triazine compound **6a** was proven by the ^1^H NMR spectrum, which exhibited distinctive singlet signals for the protons of the methyl group at *δ* 2.71 ppm and the proton of the (N–H)-thioamide group at *δ* 12.59 ppm. As well as, the coupler halogenated reagent, ethyl 4-chloro-3-oxobutanoate (4**c**) has a great affinity to undergo in situ cyclization upon coupling reaction with diazotized amine **1** through nucleophilic attack of the N–H on the acetyl group and loss of water molecule to produce the pyridopyrazolo-triazine derivative** 7** (Fig. 3). The chemical structure of triazine **7** was confirmed by its compatible analysis data. The IR spectrum displayed a specific absorption for the carbonyl group at 1736 cm^−1^. Its ^1^H NMR spectra showed a characteristic singlet at *δ* 5.77 ppm for the methylene group. Moreover, the mass analysis exhibited the molecular ion peak at *m/z* = 319 (10.96%), corresponding to a molecular formula (C_14_H_14_ClN_5_O_2_).

### DFT study

**Figure 4:**
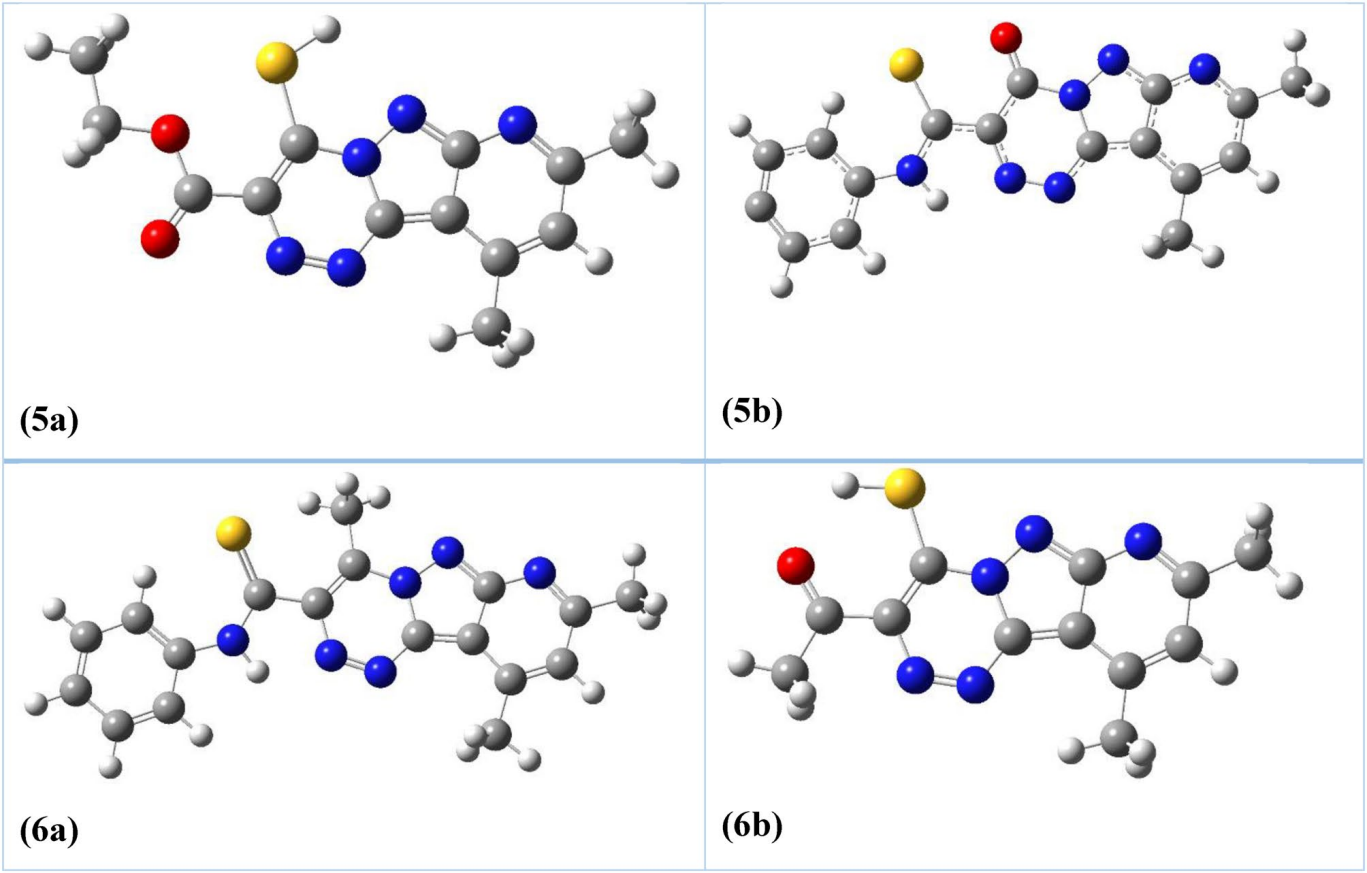
Optimized structures of compounds **5a**, **5b**, **6a**, and **6b**.

In Fig. 3, the spectroscopic technique (^1^H NMR) proved the configuration of these compounds as **5a** and **6a**, not **5b** and **6b**, and that this result can be confirmed by an investigation of the DFT and TD-DFT studies using Gaussian 09 software^32^. The optimization of the former structures was done at the B3LYP / 6-311G (d, p) level, as shown in Fig. 4. The total energy was calculated via DFT studies, which showed that the formed triazine **5a** showed a lower energy value of (− 1456.6 au) than **5b** (− 1325.4 au) indicating higher stability for **5a** than **5b**. Similarly, the produced triazine **6a** offered a lower energy value of − 1422.1 au than **6b** (− 1210 au) meaning **6a** is more stable than **6b**.

**Figure 5:**
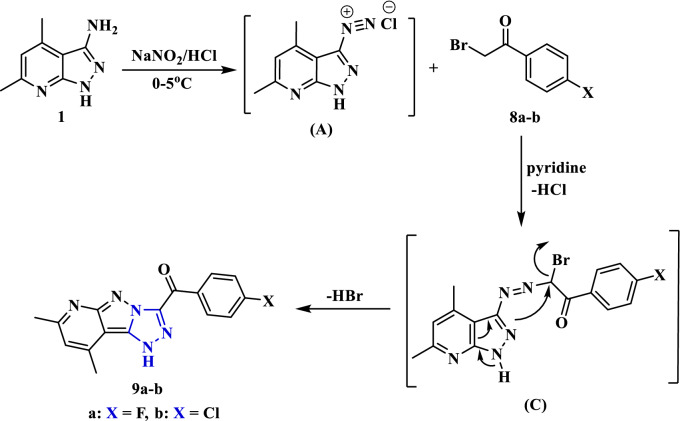
Synthesis of pyridopyrazolo-triazole derivatives **9a** and **9b**.

Treatment of the amine** 1** with sodium nitrite in the presence of concentrated HCl afforded the diazonium chloride solution **(A)**, which upon diazo-coupling with different α-bromoketones as *p*-fluorophenacyl bromide (**8a**) and *p*-chlorophenacyl bromide (**8b**) in pyridine afforded the corresponding pyridopyrazolo-triazole compounds **9a** and **9b**, respectively (Fig. 5). The triazole ring has been formed through nucleophilic substitution of bromine (intermediate **C**) by the N–H (from the pyrazole ring), as indicated by the loss of a hydrogen bromide molecule. The chemical structure of compounds **9a** and **9b** was proven by analytical analyses. The IR spectrum of compound **9a** revealed an absorption band at 3159 cm^-1^ for the N–H group. The carbonyl group was absorbed at a frequency of 1660 cm^−1^. Its ^1^H NMR spectrum displayed a characteristic singlet for the proton of the N–H group at *δ* 14.51 ppm in addition to the other expected signals that indicate the protons of the methyl, pyridine-CH, and phenylene groups.

**Figure 6:**
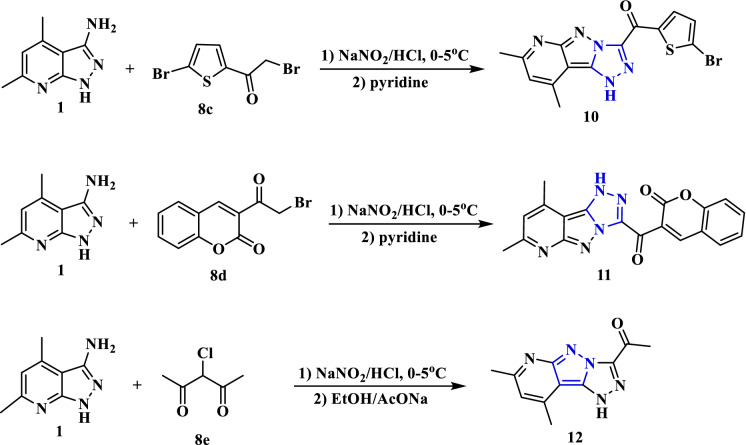
Synthesis of pyridopyrazolo-triazole derivatives **10**, **11** and **12**.

Likewise, the diazotized aminopyrazolopyridine compound was treated with heterocyclic *alpha* bromoketone derivatives **8c** and **8d** (namely; 2-bromo-1-(5-bromothiophen-2-yl)ethan-1-one and 3-(2-bromoacetyl)-2*H*-chromen-2-one) in pyridine to produce the corresponding pyridopyrazolo-triazoles **10** and **11**. According to their spectroscopy characterization the structures of pyridopyrazolo-triazoles **10** and **11** were determined. The IR spectrum of compound **10** identified absorption bands at 3349 and 1631 cm^−1^ for N–H and C=O groups. Its ^1^H NMR spectrum offered doublet signals for thiophene protons at *δ* 7.47 and 8.33 ppm and singlet for N–H proton at *δ* 14.56 ppm. The mass spectrum gave the molecular ion peak at *m/z* = 376 (17.92%), corresponding to a molecular formula (C_14_H_10_BrN_5_OS). Furthermore, the coupling reaction of 3-chloropentane-2,4-dione (**8e**) with the diazonium salt of amine **1** was carried out in the presence of ethanol and sodium acetate to afford the corresponding pyridopyrazolo-triazole derivative **12** (Fig. 6). The coupling reaction proceeded by Japp-Klingemann reaction (acetyl group cleavage) followed by intramolecular cyclization of the corresponding azo intermediate by nucleophilic substitution of the chlorine and losing hydrogen chloride molecule.

### Biological evaluation

**Figure 7:**
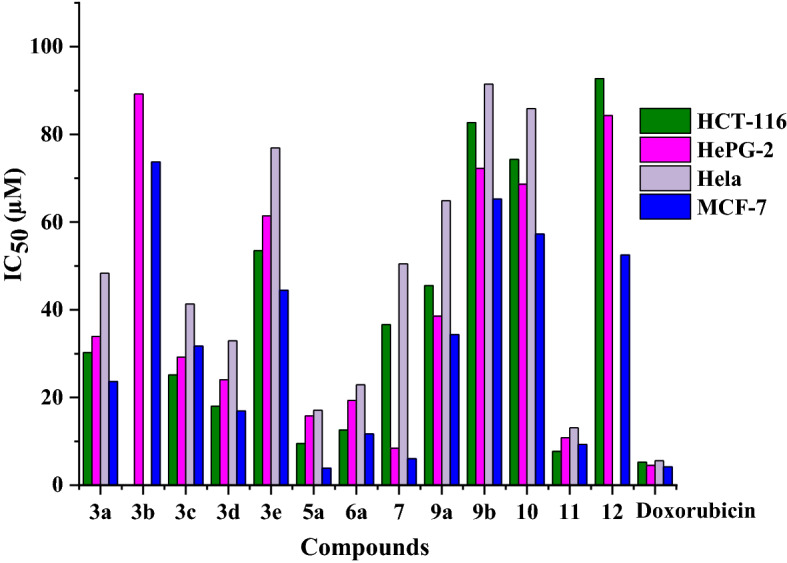
IC_50_ of the cytotoxic activity of the examined compounds against human tumor cell lines.

Accordingly, all of the newly synthesized pyridopyrazolo-triazine and triazole derivatives were examined in vitro to quantify their inhibitory activity against antitumor cell lines such as HCT-116 (Colorectal carcinoma), HEPG-2 (Hepatocellular carcinoma), Hela (Epitheliod Carcinoma Cervix cancer), and MCF-7 (Human Breast Adenocarcinoma) by using the MTT method^26,27^. Doxorubicin, one of the common small-molecule anti-cancer drugs, was employed for comparison. The concentration of compounds needed to inhibit the growth of 50% of cancer cells was expressed as (μM) and displayed in Table 1 and Fig. 7. The thirteen examined compounds showed several degrees of inhibitory effects on tested human tumor cells. Compounds **3a-e**, **5a**, **6a**, **7**, **9a**, **9b**, and **10-12** were assayed for their abilities to inhibit the HCT-116 cell line compared to doxorubicin (IC_50_ = 5.23 µM). The inhibition potency of the synthesized compounds was in the range of 7.71–92.72 µM. Triazole **11** exhibited remarkable activity with an IC_50_ value of 7.71 mM. Further, the pyridopyrazolo-triazine compounds **3d**, **5a**, and **6a** are proved to be of distinguished activity with IC_50_ values of 18.01, 9.50, and 12.58 µM, respectively. On the other hand, compounds **3a**, **3c**, **7**, **9a**, and **3e** demonstrated weak activity with IC_50_ values of 30.24, 25.14, 36.60, 45.51, and 53.49 µM, respectively. The derivatives **9b**, **10**, and **12** recorded very weak values of IC_50_ 82.68, 74.28, and 92.72 µM, respectively. The pyridopyrazolo-triazine compound **3b** showed no acceptable growth inhibitor effect. The thirteen HCT-116 inhibitors obtained were selected to be further assayed against the HePG-2 cell line and compared with doxorubicin (IC_50_ = 4.50 µM). The pyridopyrazolo-triazine compound **7** showed good cytotoxic activity (IC_50_ = 8.42 µM). Moderate cytotoxic activities were noticed by compounds **6a** and **11** with IC_50_ values of 19.32 and 10.84 µM, as well as other derivatives **3a**, **3c**, **3d**, and **9a** revealed weak antitumor effects with IC_50_ 33.94, 29.20, 24.06, and 38.53 µM. Moreover, compounds **3b**, **3e**, **5a**, **9b**, **10**, and **12** displayed low values of IC_50_ 89.22, 61.40, 51.80, 72.26, 68.67, and 84.30 µM. Also, these compounds were assayed for their abilities to inhibit the Hela cell line and compared with the reference (IC_50_ = 5.57 µM). The pyridopyrazolo-triazine derivative **3b** and the pyridopyrazolo**-**triazole **12 **appeared no acceptable cytotoxic activity on Hela cell line, compounds **5a** and **11** exhibited eminent IC_50_ values 17.09 and 13.11 µM. The moderate effect is exhibited by compounds **3a**, **3c**, **3d**, **6a**, and** 7** which recorded IC_50_ values in the range from 22.90 to 50.49 µM, the residual compounds **3e**, **9a**, **9b**, and **10** showed minimum activity with IC_50_ values of 76.93, 64.90, 91.45, and 85.90 µM, respectively. Finally, different derivatives were tested also toward the breast cell line (MCF-7). The pyridopyrazolo-triazine derivative **5a** demonstrated highly potent (IC_50_ = 3.89 µM) compared to reference drug (IC_50_ = 4.17 µM), compounds **7** and **11** gave remarkable values of IC_50_ = 6.04 and 9.29 µM, pyridopyrazolo-triazines **3d** and **6a** proved to be active with IC_50_ 16.93 and 11.71 µM, the other compounds **3a-3c**, **3e**, **9a**, **9b**, **10**, and **12** registered IC_50_ in the range 23.64 to 73.74 µM. From previous data we found the MCF-7 among the examined cells exhibited the best growth inhibitor, but the Hela offered the least activity.

**Table 1:** Cytotoxic activity of synthesized and designed compounds against human tumor cell lines.

Compounds	In vitro Cytotoxicity IC_50_ ± S. D (µM)			
	HCT-116	HePG-2	Hela	MCF-7
**3a**	30.24 ± 2.30	33.94 ± 2.43	48.33 ± 2.70	23.64 ± 1.84
**3b**	> 100	89.22 ± 4.61	> 100	73.74 ± 4.02
**3c**	25.14 ± 2.14	29.20 ± 2.26	41.32 ± 2.41	31.71 ± 2.26
**3d**	18.01 ± 1.42	24.06 ± 2.10	32.95 ± 2.20	16.93 ± 1.30
**3e**	53.49 ± 3.11	61.40 ± 3.52	76.93 ± 3.62	44.45 ± 2.71
**5a**	9.50 ± 0.83	15.80 ± 1.30	17.09 ± 1.43	3.89 ± 0.28
**6a**	12.58 ± 1.02	19.32 ± 1.51	22.90 ± 1.80	11.71 ± 0.92
**7**	36.60 ± 2.51	8.42 ± 0.70	50.49 ± 2.91	6.04 ± 0.50
**9a**	45.51 ± 2.80	38.53 ± 2.62	64.90 ± 3.36	34.33 ± 2.32
**9b**	82.68 ± 4.21	72.26 ± 4.00	91.45 ± 4.63	65.27 ± 3.71
**10**	74.28 ± 3.84	68.67 ± 3.74	85.90 ± 4.20	57.26 ± 3.30
**11**	7.71 ± 0.62	10.84 ± 0.90	13.11 ± 1.01	9.29 ± 0.73
**12**	92.72 ± 4.70	84.30 ± 4.32	> 100	52.51 ± 2.92
Doxorubicin	5.23 ± 0.33	4.50 ± 0.20	5.57 ± 0.46	4.17 ± 0.20

### Molecular modeling analysis

Molecular docking techniques are used to calculate chemical and surface properties to investigate thermodynamics of biological systems. The docking scores, bond distances, and interactions of the ligands with particular amino acids were displayed in Table 2. It was found from tabulated results these compounds under study showed perfect fitting inside the active site of the protein and gave scores of binding energy in the range from − 6.0161 to − 7.8182 kcal/mol.

**Table 2:** Predictive docking scores and particular interactions of the ligands and the protein.

Cpd. No	Binding energy (S) Kcal/mol	RMSD	Distance (Å)	Binding interactions		
				Ligand	Receptor	Interaction type
**3a**	− 6.8678	1.3586	3.37	*N*-atom of pyridine ring	Asn 575	H–acceptor
			3.38	*N*-atom of pyrazole-ring	Lys 501	H–acceptor
			3.75	Pyrazole-ring	Tyr 472	π–π
			3.98	Triazine‐ring	Phe 480	π–π
			3.78	Triazine‐ring	Tyr 472	π–π
**3b**	− 6.4952	0.8897	3.28	*N*-atom of pyridine ring	His 483	H–acceptor
			4.29	Methyl (CH_3_)	Phe 480	H–π
**3c**	− 6.3936	1.1003	3.39	*N*-atom of pyridine ring	His 483	H–acceptor
			3.67	Methyl (CH_3_)	Tyr 472	H–π
			4.22	Methyl (CH_3_)	Phe 480	H–π
			4.67	Pyridine‐ring	His 571	π–H
**3d**	− 6.7661	1.0013	3.4	*N*-atom of amid group	Leu 536	H–donor
			4.3	Triazine‐ring	Cys 481	π–H
			3.76	Pyridine‐ring	His 483	π–π
**3e**	− 6.6978	1.0001	3.26	*N*-atom of pyridine ring	His 483	H–acceptor
			3.17	*O*-atom of methoxy	Gln 75	H–acceptor
			4.18	Methyl (CH_3_)	Phe 480	H–π
**5a**	− 7.7362	0.7456	3.51	*N*-atom of pyridine ring	Lys 501	H–acceptor
			3.51	*N*-atom of pyrazole ring	Lys 501	H–acceptor
			2.84	*O*-atom of carbonyl (CO)	His 483	H–acceptor
			3.21	*O*-atom of carbonyl (CO)	His 571	H–acceptor
			3.75	Pyrazole‐ring	Tyr 472	π–π
			3.91	Triazine-ring	Phe 480	π–π
**6a**	− 7.8182	0.8162	3.64	*N*-atom of pyridine ring	Asn 575	H–acceptor
			3.22	*S*-atom of thioamide group	His 571	H–acceptor
			3.83	Pyrazole‐ring	Tyr 472	π–π
**7**	− 7.2146	0.6854	3.01	Cl-atom	Asn 493	H–acceptor
			3.11	*O*-atom of carbonyl	His 483	H–acceptor
			2.95	*O*-atom of carbonyl Pyrazole‐ring	His 571	H–acceptor
			3.9		Tyr 472	π–π
**9a**	− 6.0161	1.4017	3.25	*N*-atom of pyridine ring	Asn 493	H–acceptor
			3.5	*O*-atom of carbonyl Phenyl‐ring	Lys 501	H–acceptor
			4.17	Pyridine‐ring	Phe 480	π–H
			4.48	Pyrazole‐ring	His 571	π–H
			3.99		Tyr 472	π–π
**9b**	− 6.3226	1.1326	2.93	*O*-atom of carbonyl	Lys 501	H–acceptor
			4.15	Phenyl‐ring	Phe 480	π–H
			3.83	Triazole‐ring	Tyr 472	π–π
			3.63	Pyridine‐ring	His 483	π–π
**10**	− 6.1633	1.4107	3.26	*N*-atom of pyridine ring	His 483	H–acceptor
			3.78	Methyl (CH_3_)	Tyr 472	H–π
			3.54	Triazole‐ring	Phe 480	π–H
**11**	− 7.2031	0.7554	3.07	*O*-atom of carbonyl	Asn 493	H–acceptor
			3.34	O-atom of 2-Pyranone	Lys 501	H–acceptor
			3.85	2-Pyranone‐ring	Phe 480	π–π
			3.67	2-Pyranone‐ring	Tyr 472	π–π
**12**	− 6.5458	1.4461	3.44	*N*-atom of pyridine ring	Lys 501	H–acceptor
			3.86	*N*-atom of pyrazole-ring	Lys 501	H–acceptor
			3.73	Pyrazole‐ring	Tyr 472	π–π
			3.82	Triazole‐ring	Phe 480	π–π
Doxorubicin	− 7.8585	1.3316	3	*O*-atom of hydroxyl	Tyr 409	H–acceptor
			3.94	Phenyl-ring	Tyr 472	π–π

**Figure 8:**
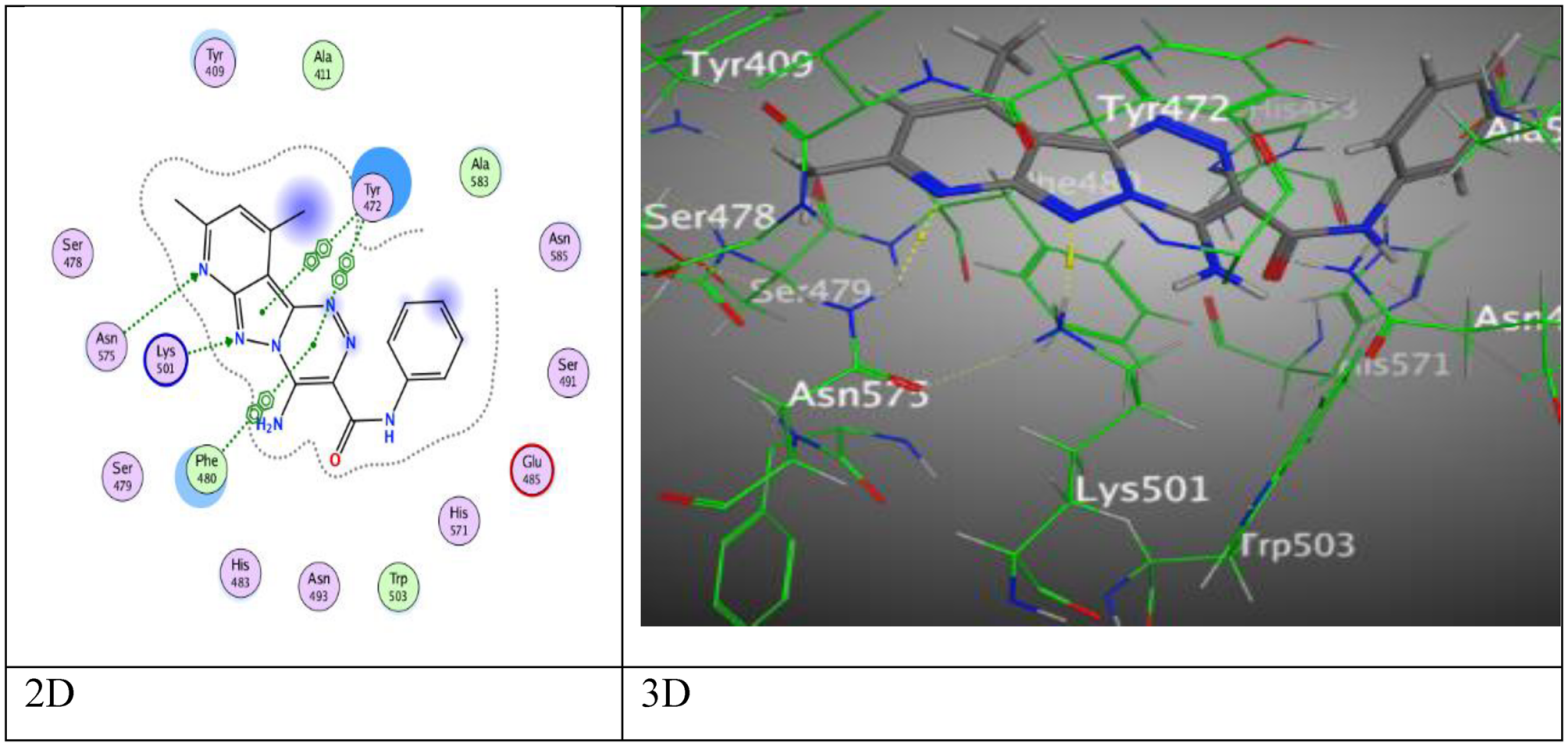
The binding interactions of compound **3a** with active sites of (PDB ID: 5IVE).

Particular binding score (S = − 6.8678 kcal/mol) of pyridopyrazolo-triazine **3a** came from five intermolecular attractions, two hydrogen acceptor-bonds through pyridine ring via N-atom represented attraction with Asn 575 through (3.37 Å), and N-atom of pyrazolyl ring that binding with Lys 501 at an intermolecular distance 3.38 Å, three π–π interactions, two π–π interactions were revealed between Tyr 472 with both of pyrazolyl and triazine rings through intermolecular distance (3.75, 3.78 Å), one π–π interaction occurred between triazine ring and Phe 480 (3.98 Å) (Fig. 8).

While, pyridopyrazolo-triazine **3b** containing chlorine atom in Fig. S40 displayed two types of interactions, hydrogen acceptor-bond between the N-atom of pyridine moiety and His 483 through intermolecular distance 3.28 Å, H–π interaction between one of the methyl group on the pyridine ring and Phe 480 amino acid of 5IVE (4.29 Å), through binding energy score (S = − 6.4952) kcal/mol. Moreover, the molecular docking of pyridopyrazolo-triazine have methyl substituent **3c** with active site of protein; presented four interactions, hydrogen acceptor-bond for N-atom of pyridine ring with His 483 (3.39 Å), and other three H-π interactions were presented through two bindings from methyl group of pyridine with Tyr 472 and Phe 480 (3.67, 4.22 Å), respectively and last was formed from pyridine ring with His 571 by an intermolecular distance 4.67 Å over binding score (S = − 6.3936 kcal/mol) (Fig. S41).

**Figure 9:**
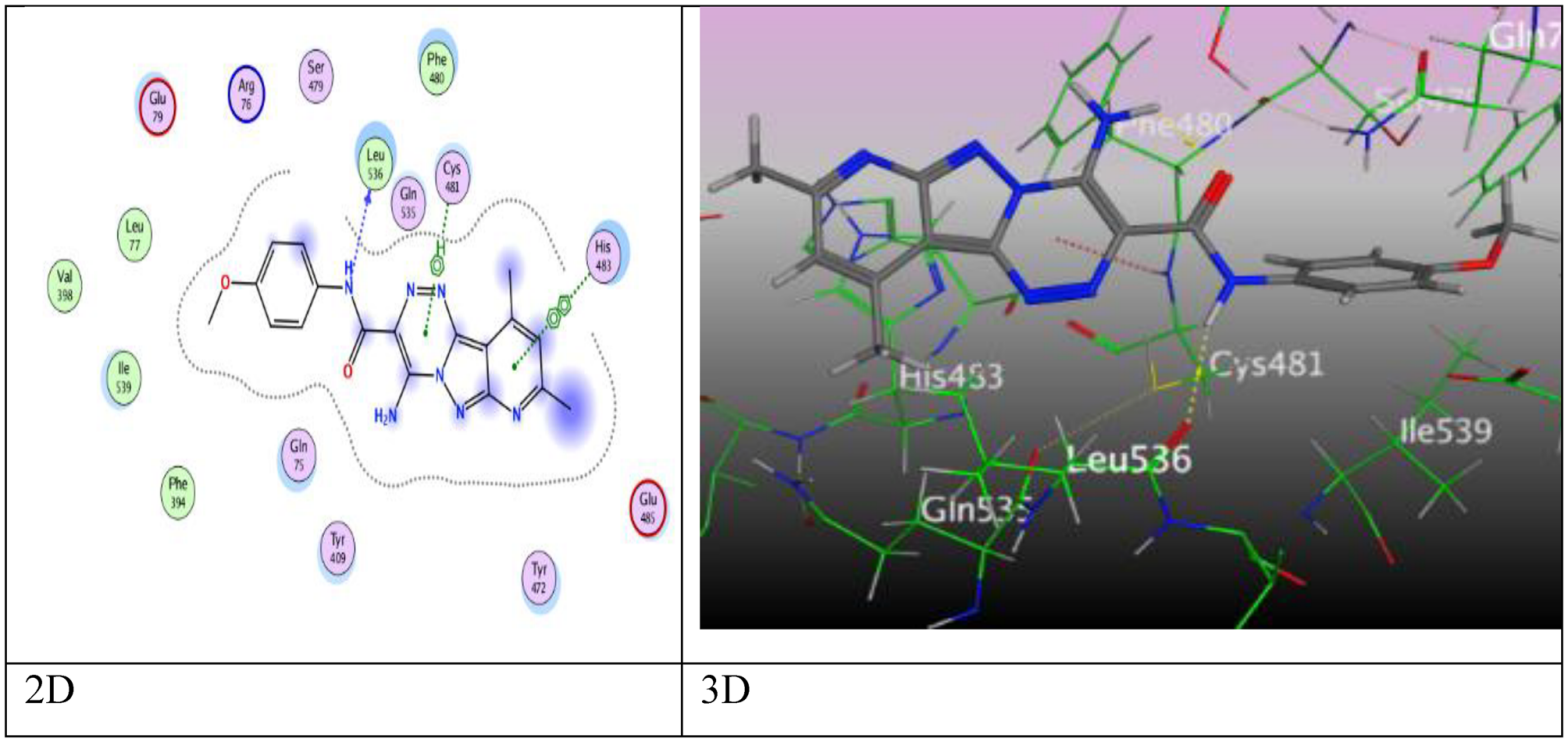
The binding interactions of compound **3d** with active sites of (PDB ID: 5IVE).

Further, pyridopyrazolo-triazine containing methoxy group **3d** exhibited an energy score (S = − 6.7661 kcal/mol) and demonstrated hydrogen donor-bond (3.40 Å) arisen from nitrogen-atom of amide group to O-atom of Leu 536, π–H interaction between triazine and N atom Cys 481, and π–π interaction between pyridine ring with His 483 through an intermolecular distance (4.30 and 3.76 Å), respectively (Fig. 9).

**Figure 10:**
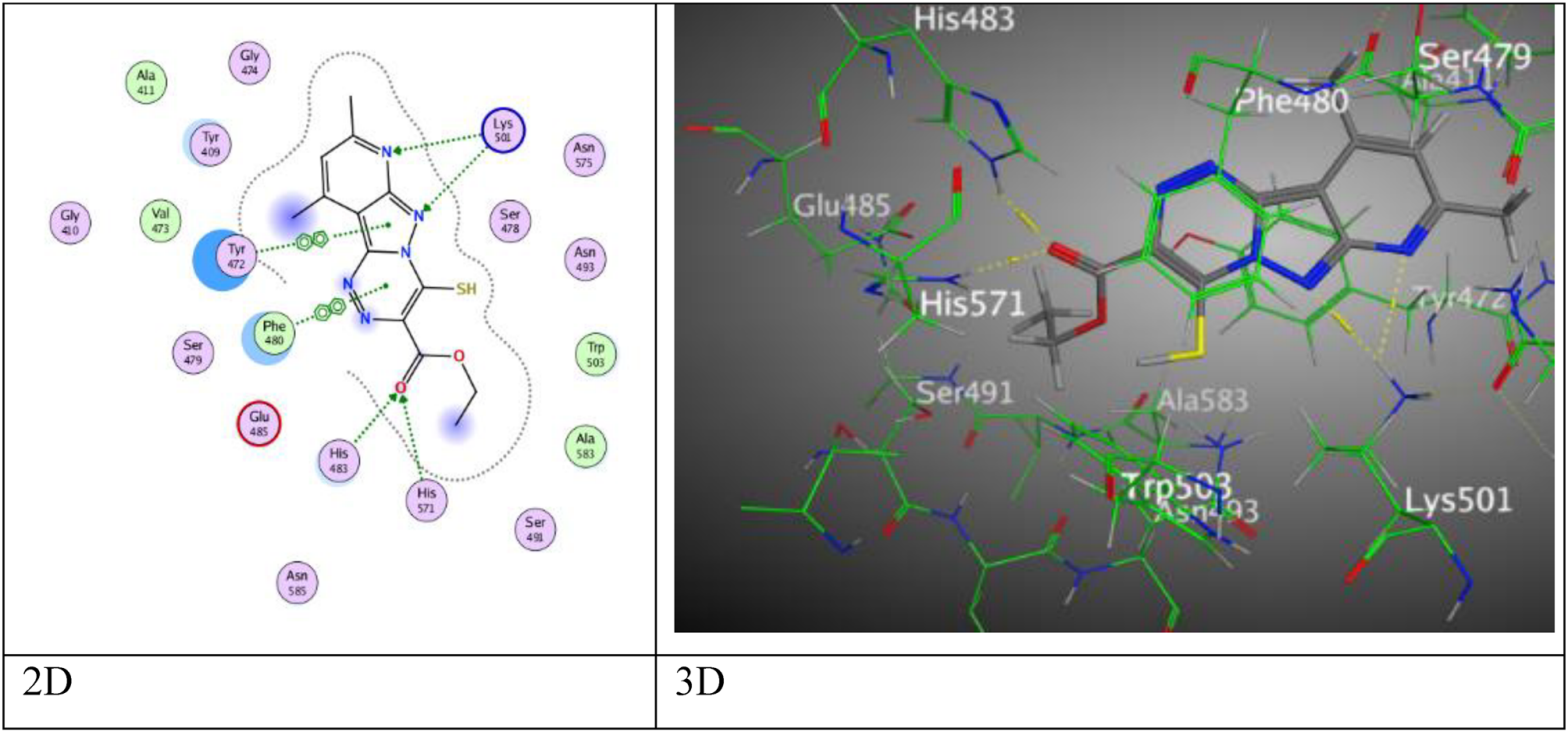
The binding interactions of compound **5a** with active sites of (PDB ID: 5IVE).

Through the docking process, pyridopyrazolo-triazine with acetyl group **3e** displayed three interaction, two hydrogen bonds between nitrogen-atom of pyridine ring with His 483, and O-atom of acetyl group with Gln 75 (3.26 Å and 3.17 Å), and H-π interaction between C-atom of methyl group of pyridine with Phe 480 (4.18 Å) (Fig. S42). Furthermore, pyridopyrazolo-triazine **5a** gave a good binding energy score (S = − 7.7362  kcal/mol) over six attractions, two H-bonds occurred between N-atom of pyridine and pyrazolyl ring with the same amino acid Lys 501 through the same intermolecular distance (3.51 Å), other two H-bonds formed between O-atom of carbonyl ester with His 483 and His 571 through distances (2.84, 3.21 Å), respectively. The fifth and sixth attractions appeared as π–π interaction among pyrazolyl ring and triazine with Tyr 472 and Phe 480 (3.75, 3.91 Å) (Fig. 10).

**Figure 11:**
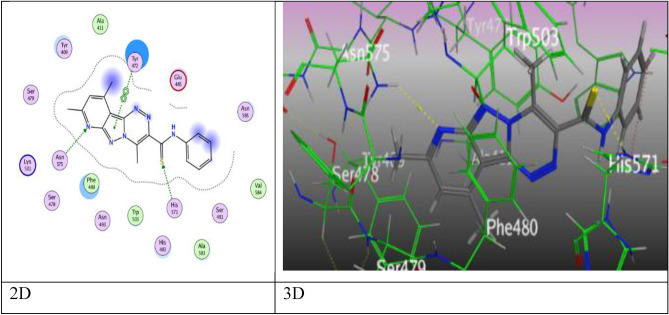
The binding interactions of compound **6a** with active sites of (PDB ID: 5IVE).

After a while, pyridopyrazolo-triazine derivative **6a** in Fig. 11, showed an eminent binding score (S = − 7.8182 kcal/mol) came from three intermolecular attractions, two hydrogen acceptor-bond occurred among N-atom of pyridine ring with Asn 575, S-atom of thioamide moiety and His 571 through an intermolecular distance (3.64 Å and 3.22 Å), respectively, in addition to one π–π interaction between pyrazolyl ring and Tyr 472 (3.83 Å).

**Figure 12:**
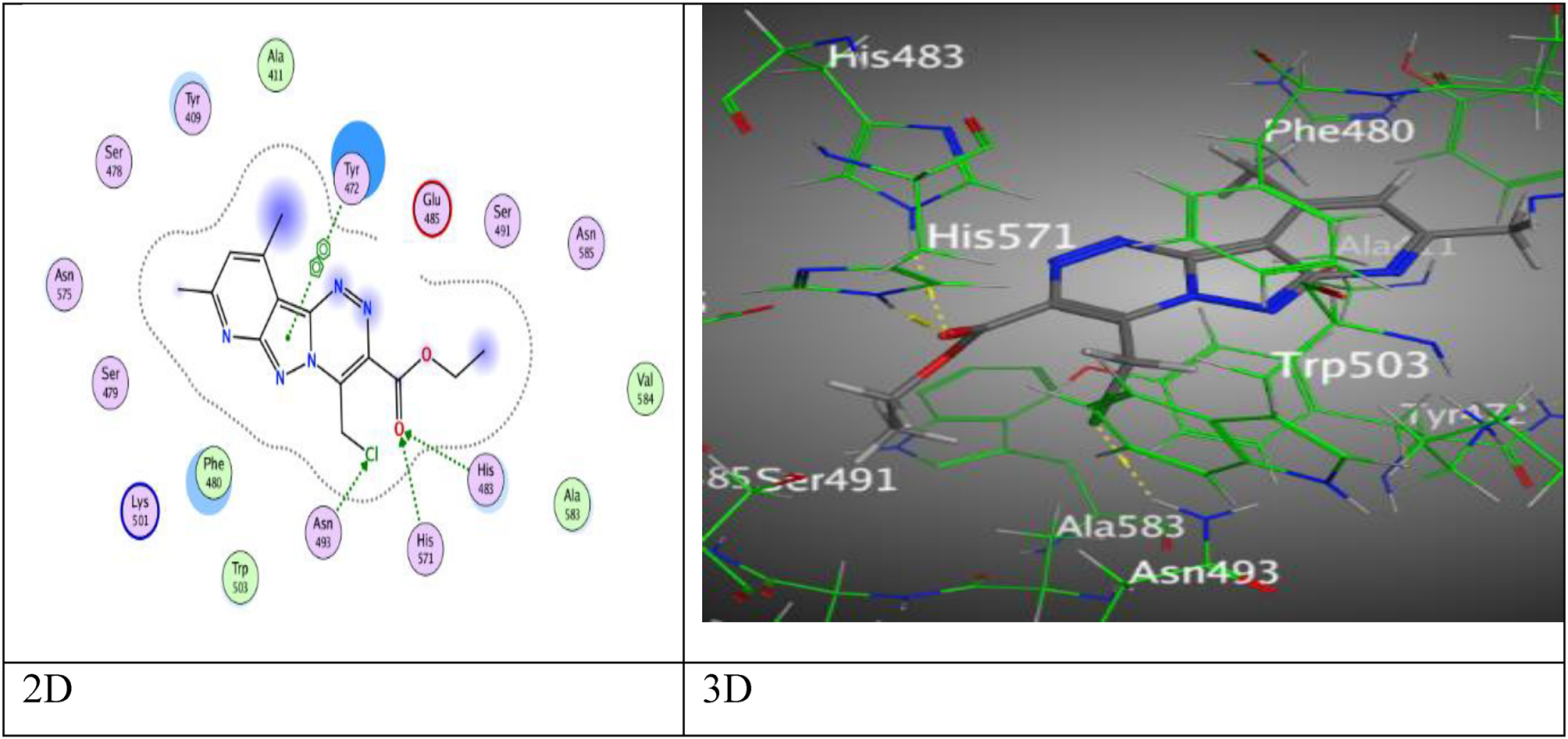
The binding interactions of compound** 7** with active sites of (PDB ID: 5IVE).

Then, compound **7** has respectable binding energy (− 7.2146 kcal/mol). Its interested active sites were displayed three H-bond acceptor, the first was raised between chlorine-atom with Asn 493 (3.01 Å), and two interactions from O-atom of carbonyl group with His 483 and His 571 by an intermolecular distances (3.11 and 2.95 Å), while the fourth attraction is π–π bond between pyrazolyl ring with Tyr 472 (3.90 Å) (Fig. 12).

Where, pyridopyrazolo-triazole containing fluorine **9a** displayed five attractions, two H-bonds were occurred as following: one bond between N-atom of pyridine ring with Asn 493, one bond between oxygen-atom of ketonic group with Lys 501, two π–H interactions between both six rings (phenyl and pyridine rings) with Phe 480 and His 571, and the last π–π interaction among pyrazolyl ring with Tyr 472, these interactions happened at different intermolecular distances as (3.25, 3.50, 4.17, 4.48, and 3.99 Å), respectively in (Fig. S43). Otherwise, pyridopyrazolo-triazole substituted with chlorine-atom **9b** revealed four attractions, a H-acceptor bond at intermolecular distance (2.93 Å) between oxygen-atom of carbonyl group (C=O) with Lys 501, π–H interaction between benzene ring with Phe 480, and two π–π interactions which created between both rings of triazole and pyridine with Tyr 472 and His 483 respectively, at various intermolecular distances 4.15 Å, 3.83 Å, and 3.63 Å (Fig. S44). Meanwhile, pyridopyrazolo-triazole **10** demonstrated hydrogen- acceptor bond between N-atom of pyridine ring with His 483 (3.26 Å), two different intermolecular distances (3.78 Å and 3.54 Å), respectively, manifest for two H-π interactions between methyl group of pyridine moiety with Tyr 472 and the another between triazole moiety with Phe 480 in Fig. S45 through binding score (S = − 6.1633 kcal/mol).

**Figure 13:**
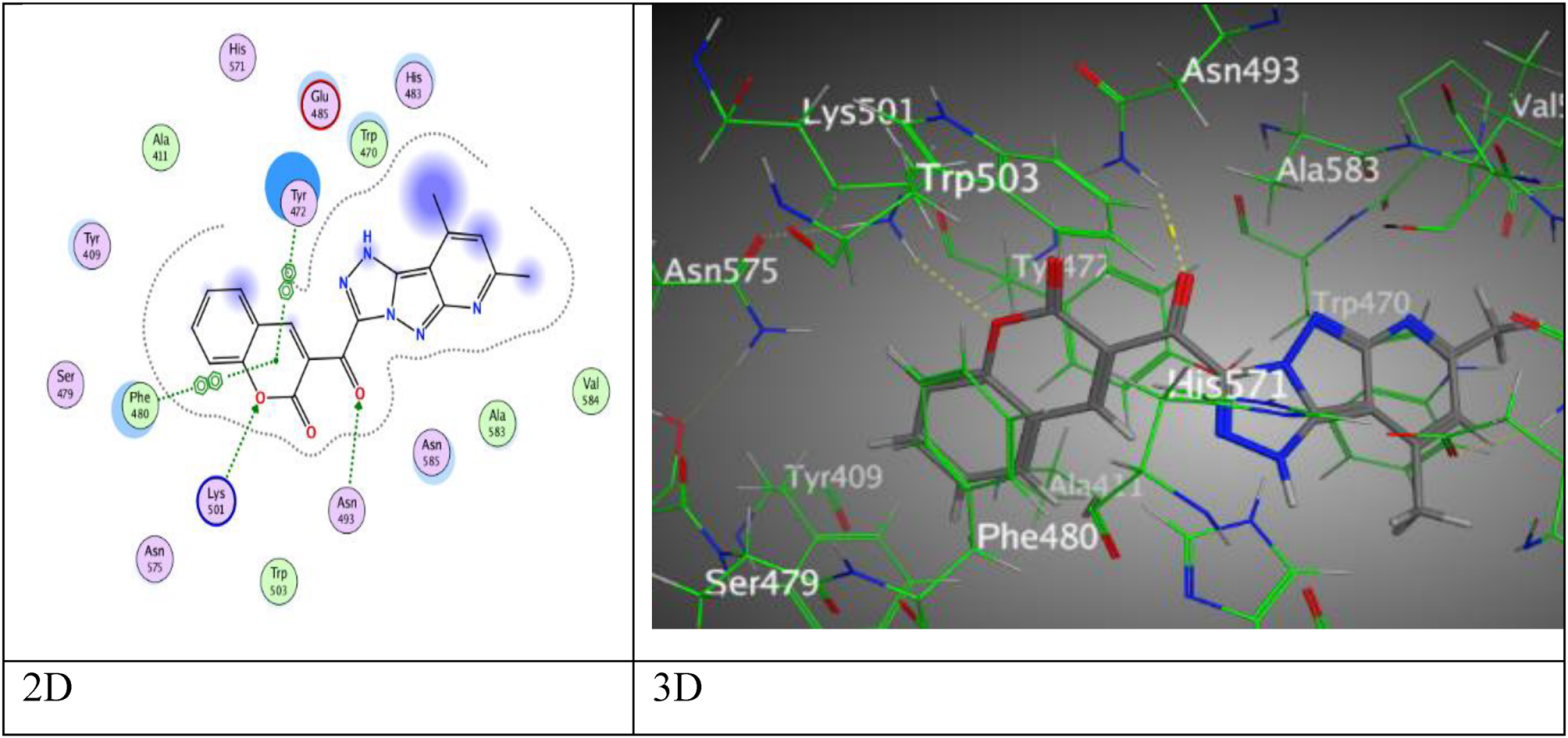
The binding interactions of compound **11** with active sites of (PDB ID: 5IVE).

**Figure 14:**
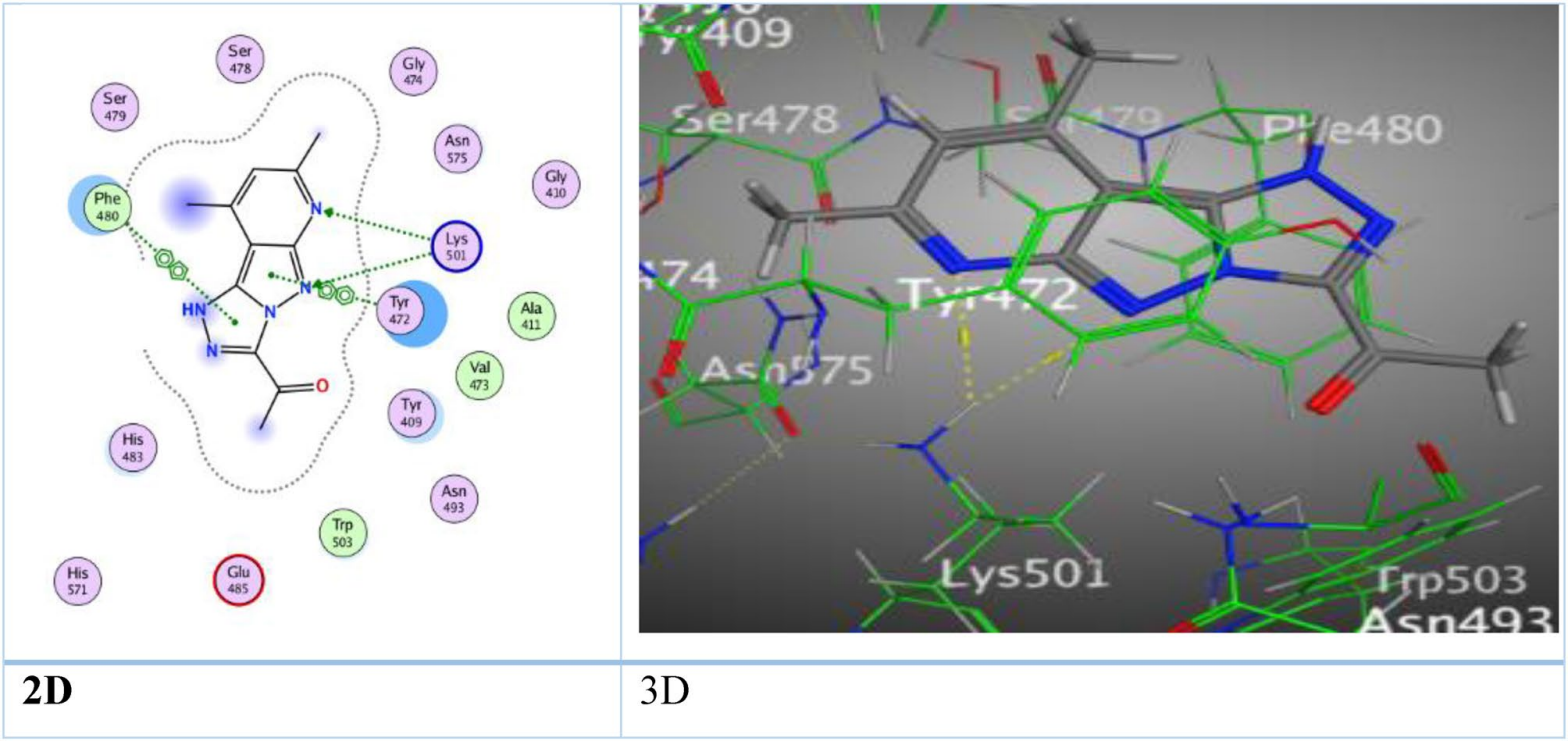
The binding interactions of compound **12** with active sites of (PDB ID: 5IVE).

Finally, pyridopyrazolo-triazole derivatives **11** and **12** were displayed four intermolecular attractions, derivative **11** showed two H-bonds through O-atom of carbonyl group and coumarin unit with Asn 493 and Lys 501 (3.07 and 3.34 Å), respectively, two π–π interactions via Pyran-2-one ring of coumarin with Phe 480 and Tyr 472 at an intermolecular distances (3.85 Å and 3.67 Å), respectively by proper binding score (S = ‐7.2031 kcal/mol). Meanwhile, derivative **12** revealed two H-acceptor bonds between Lys 501 with N-atom of pyridine and pyrazolyl rings (3.44 and 3.86 Å), respectively, two π–π interactions, one π–π interaction was revealed between pyrazole ring with Tyr 472, and one through triazole ring with Phe 480 at an intermolecular distances (3.73, 3.82 Å), respectively (Figs. 13, 14).

Reference compound for the docking is doxorubicin. It was docked with (PDB ID: 5IVE) and demonstrated different interactions as following: H-acceptor bond between O-atom with Tyr 409 (3.00 Å), in addition to pi–pi interaction among phenyl ring and Tyr 472 (3.94 Å) through proper binding score (S = − 7.8585 kcal/mol) as shown in Fig. S46.

### ADMET prediction

Drug-likeness was predicted using Lipinski's rule of five to investigate the physicochemical parameters of the newly synthesized compounds^33^. The rule allows only one violation of the criteria^34,35^. The physicochemical properties of the most active compounds **5a**, **6a**, **7**, and **11** were done via Swiss ADME prediction website (https://www.swiss adme.ch). These compounds had zero violation so they obeyed Lipinski's rule of five and could act as oral drugs as showed in Table 3.

**Table 3:** Important computed parameters of Lipinski's rule of five, its violation and drug-likeness of the most potent compounds **5a**, **6a**, **7**, and **11**.

Compounds	MW	M log P	HBA	HBD	TPSA (Å^2^)	*n*RB	*n*Vs	Drug-likeness
5a	303.34	1.98	6	0	121.07	3	0	Yes
6a	348.42	2.99	4	1	100.09	3	0	Yes
7	319.75	2.50	6	0	82.27	4	0	Yes
11	359.34	1.98	6	1	106.15	2	0	Yes

*MW* molecular weight ≤ 500, *LogP* logarithm of partition coefficient between n-octanol and water ≤ 5, *HBA* hydrogen-bonded acceptor ≤ 10, *HBD* hydrogen-bonded donor ≤ 5, *TPSA* topological polar surface area ≤ 140, *nRB* number of rotatable bonds ≤ 10, *nVs* number of violation from lipinski’s rule of five ≤ 1.

### Structure activity relationships (SAR)

The pyrazolopyridine hetero-nuclei were fused to rings triazine and triazole, the size of the ring and the presence of different substituents seemed to affect the magnitude of activity^36,37^. The pyridopyrazolo-triazine that attached to the acetamide unit, the analogue **3d** recorded specific activities with IC_50_ = 18.01, 24.06, 32.95, and 16.93 µM, respectively toward four tested cell lines compared to the residual analogues **3a-3c** and **3e**, that is due to presence a strong electron-donating group (methoxy group) on the phenyl ring of the cyanoacrylmide^38,39^. Moreover, triazine **5a** showed good growth inhibitor with values of IC_50_ = 9.50, 17.09, and 3.89 µM toward HCT-116, Hela, and breast cell lines unlike **6a** and **7** because it has different substituents -SH group at position 4 in addition to the ester group on the triazine **5a** and that enhanced the cytotoxic activity^40^. While triazine **7** containing on chloromethyl (–CH_2_Cl) has the highest potency with respectable IC_50_ value 8.42 µM against HePG-2 cell line. Triazole system **11** containing coumarin substituted exhibited acceptable breast, colon, and Hela anticancer properties more than other triazoles, and also it found coumarin moiety has significant antitumor activities^41–45^, and it gave IC_50_ = 7.71, 10.84, 13.11, and 9.29 µM against all examined cell lines. The previous results were confirmed by molecular docking study, compounds **5a**, **6a**, **7**, and **11** demonstrated highly binding energy **(S = **− 7.8182, − 7.7362, − 7.2146, and − 7.2031 kcal/mol) through acceptor hydrogen bonds and *pi*-*pi* interactions with active sites of several residues such as Lys 501, His 483, His 571, Tyr 472, Phe 480, Asn 575, and Asn 493.

## Conclusion

We have felicitously prepared a series of pyridopyrazolo-triazine and pyridopyrazolo-triazole via simple conditions of coupling reaction of diazonium salt of amino pyrazolopyridine with active methylene of cyanoacetamide, halogenated reagents, and *alpha* halo ketone derivatives. These new derivatives were examined against different cell lines (HCT-116, HepG2, MCF-7, and Hela). Interestingly, the pyrazolopyridine analogues **3b and 12** showed less activity toward colorectal and Hela cell lines. Pyridopyrazolo-triazine **5a** was the most active derivative for the MCF-7 cancer cell line and gave the special value of IC_50_ = 3.89 µM compared to the reference drug. The pyridopyrazolo-triazine derivative **6a** gave good activity, with IC_50_ = 12.58 and 11.71 µM, respectively, toward HCT-116 and MCF-7 cell lines. The pyridopyrazolo-triazine **7** showed remarkable activity against the Hep-G2 cell line with IC_50_ = 8.42 µM. The triazole **11** demonstrated distinct IC_50 values of_ 7.71 and 13.11 µM, respectively, against both the tested HCT-116 and Hela carcinoma cell lines. There is agreement between the theoretically predicted and experimentally obtained results, where compounds **5a**, **6a**, **7**, and **11** showed highly biological activities and also exhibited an eminent free energy score. The binding energy score of pyridopyrazolo derivatives, which came through H-bonds, *pi*–*pi*, and *pi*–H interactions with the suitable receptors, follows the order 6a > 5a > 7 > 11 with S = − 7.8182, − 7.7362, − 7.2146, and − 7.2031 kcal/mol, respectively.

## Supplementary Information

Supplementary Information.

## Data Availability

The datasets used and/or analyzed during the current study available from the corresponding author on reasonable request.
