# Supplementary material for: Anticancer evaluation and molecular docking of new pyridopyrazolo-triazine and pyridopyrazolo-triazole derivatives
Source: Sci Rep. 2023 Feb 16;13:2782. doi: 10.1038/s41598-023-29908-y (PMC9935538; doi:10.1038/s41598-023-29908-y)
Supplement: Supplementary file 1 — Supplementary Information. [file 41598_2023_29908_MOESM1_ESM.docx]

**Anticancer evaluation and molecular docking of new pyridopyrazolo-triazine and pyridopyrazolo-triazole derivatives**

Mohamed R. Elmorsy^1🖂^, Ehab Abdel-Latif^1^, Hatem E. Gaffer^2^, Samar E. Mahmoud^1^ & Ahmed A. Fadda^1^

*^1^Department of Chemistry, Faculty of Science, Mansoura University, 35516 Mansoura, Egypt.*

*^2^Dyeing, Printing and Auxiliaries Department, National Research Centre, 12622 Cairo, Egypt.*

*^🖂^Corresponding author. E-mail address: m.r.elmorsy@gmail.com*

**1. Spectral analysis**


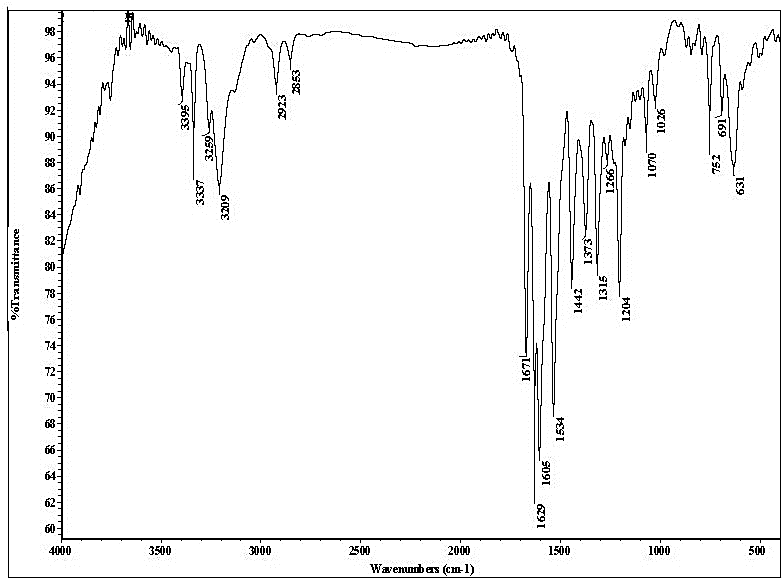

**Figure (S1): IR spectrum of pyridopyrazolo-triazine compound (3a)**

**
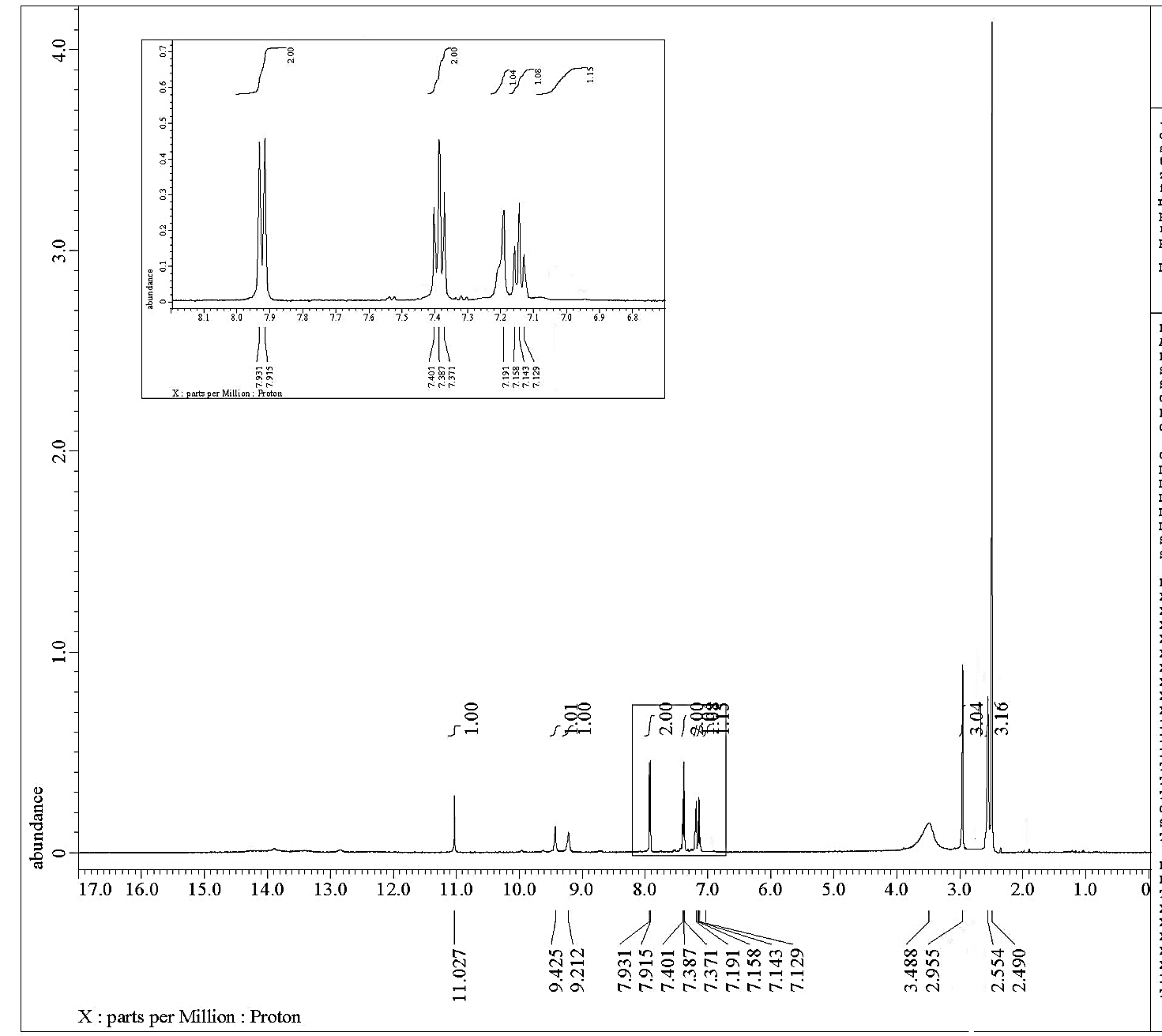
**

**Figure (S2): ^1^H NMR spectrum of pyridopyrazolo-triazine compound (3a)**

**Figure (S3): Mass spectrum of pyridopyrazolo-triazine compound (3a)**

**
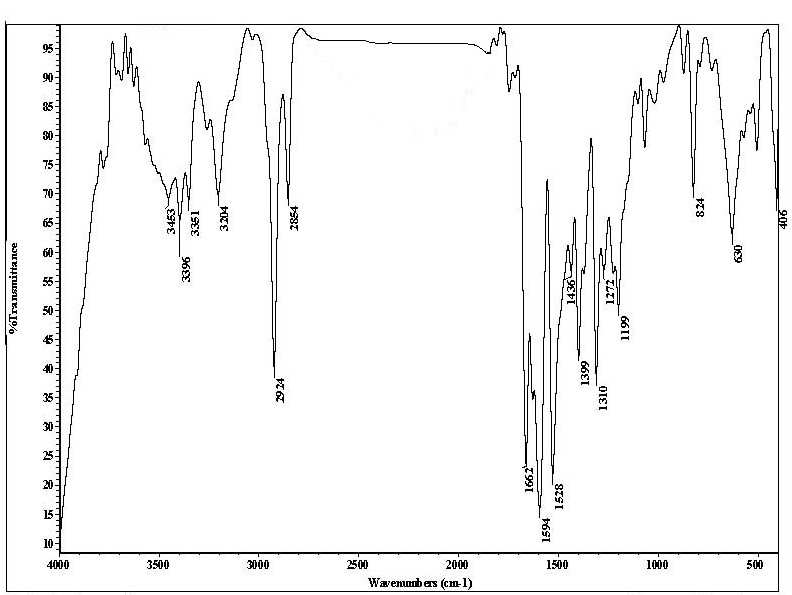
**

**Figure (S4): IR spectrum of pyridopyrazolo-triazine compound (3b)**

**
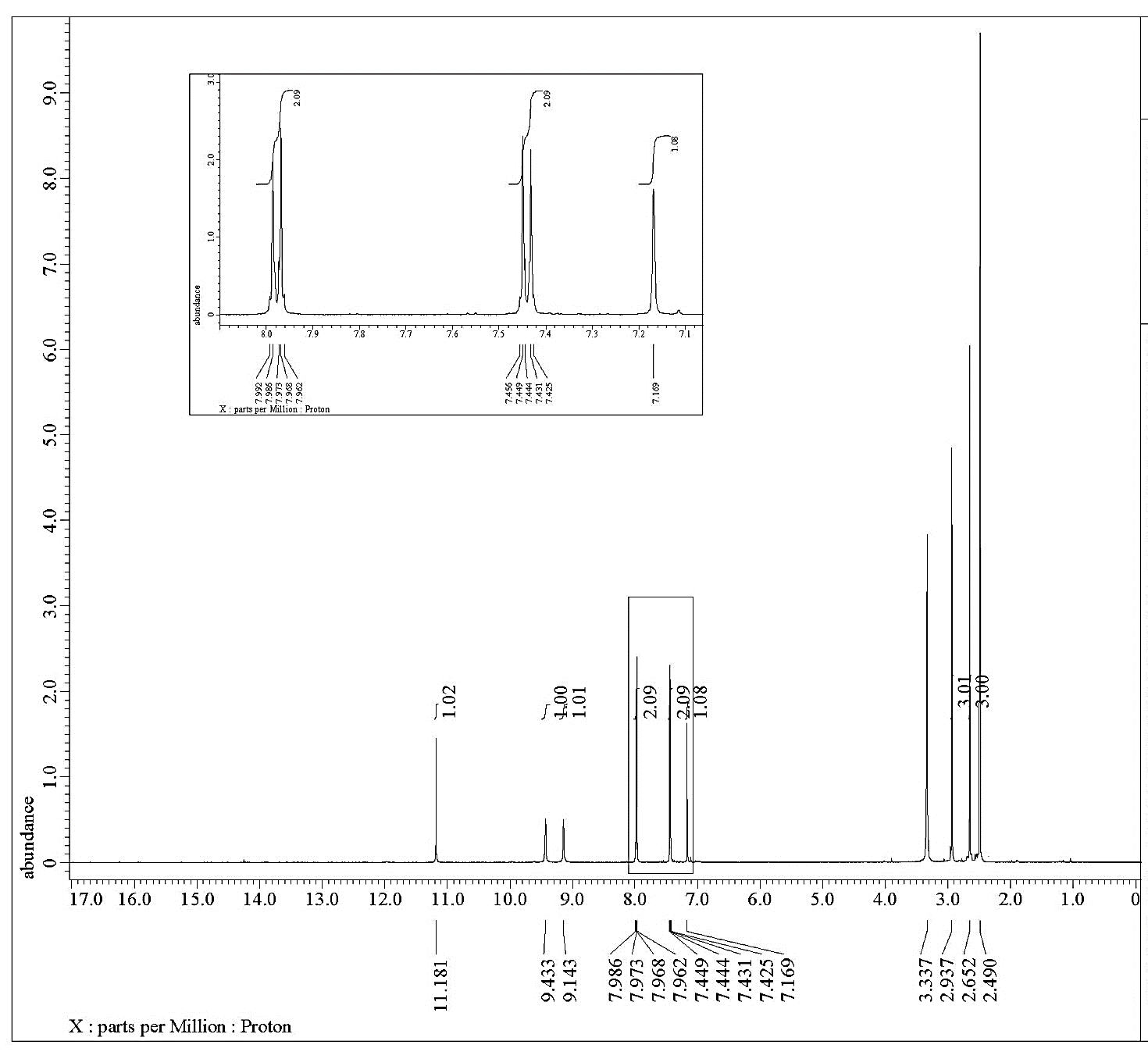
**

**Figure (S5): ^1^H NMR spectrum of pyridopyrazolo-triazine compound (3b)**

**Figure (S6): Mass spectrum of pyridopyrazolo-triazine compound (3b)**

**
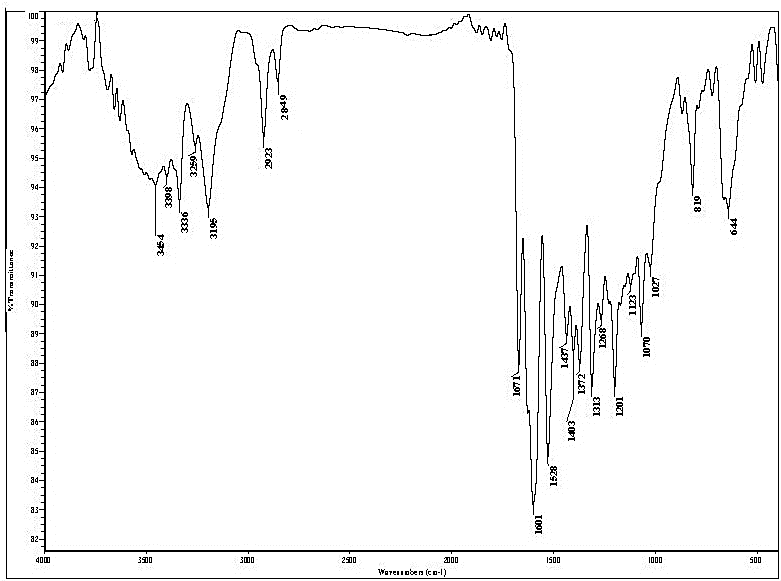
**

**Figure (S7): IR spectrum of pyridopyrazolo-triazine compound (3c)**

**
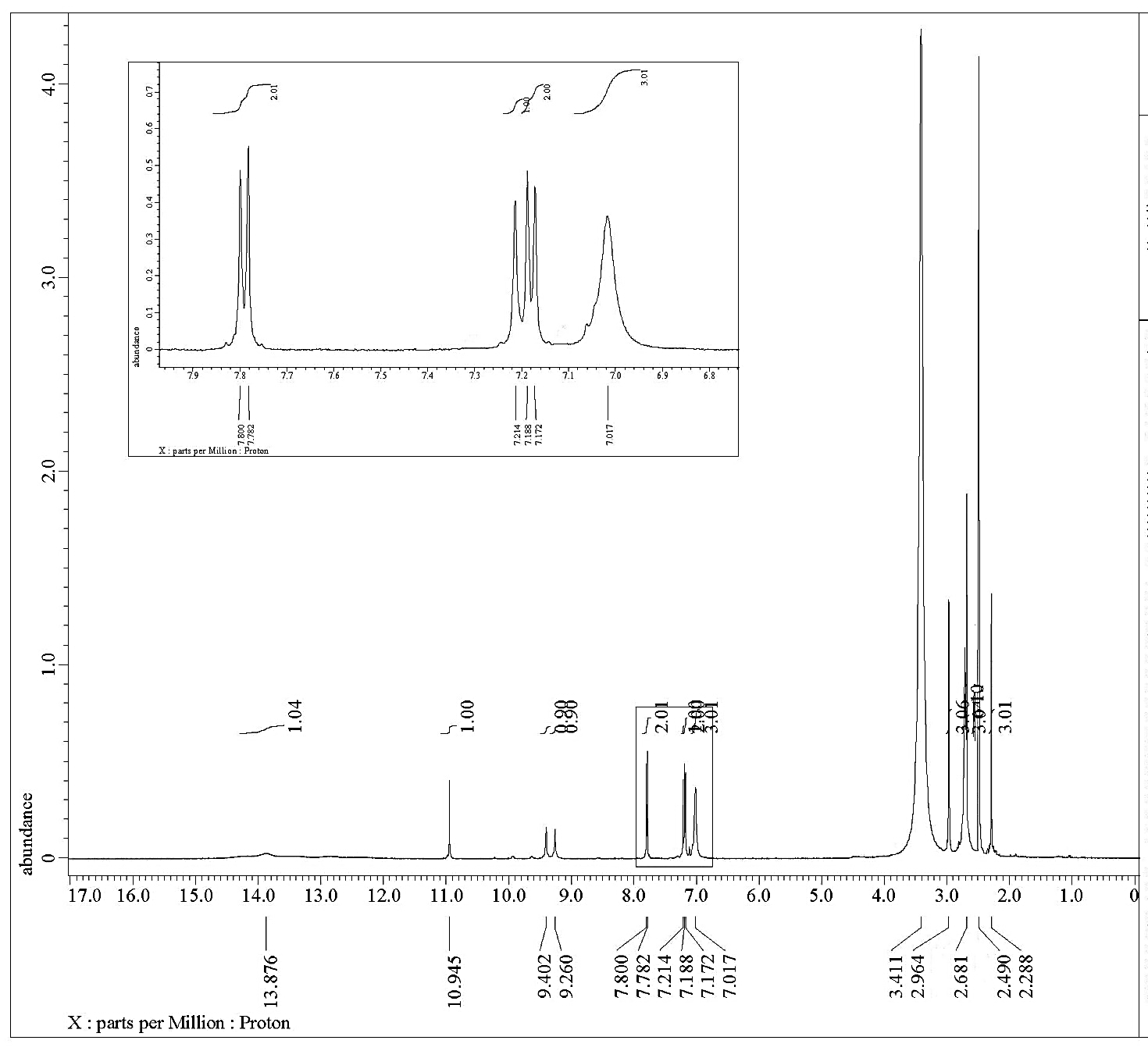
**

**Figure (S8): ^1^H NMR spectrum of pyridopyrazolo-triazine compound (3c)**

**Figure (S9): Mass spectrum of pyridopyrazolo-triazine compound (3c)**

**
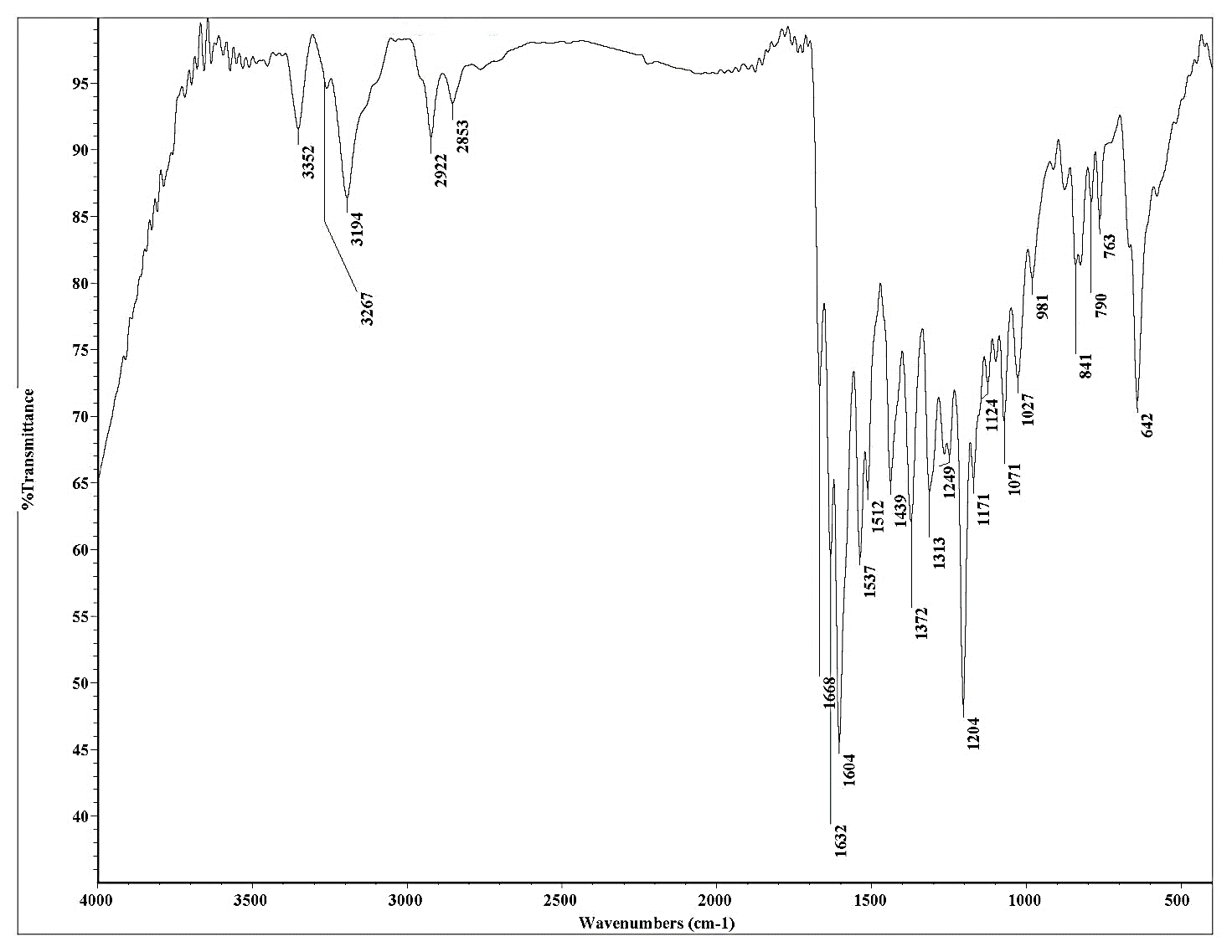
**

**Figure (S10): IR spectrum of pyridopyrazolo-triazine compound (3d)**

**
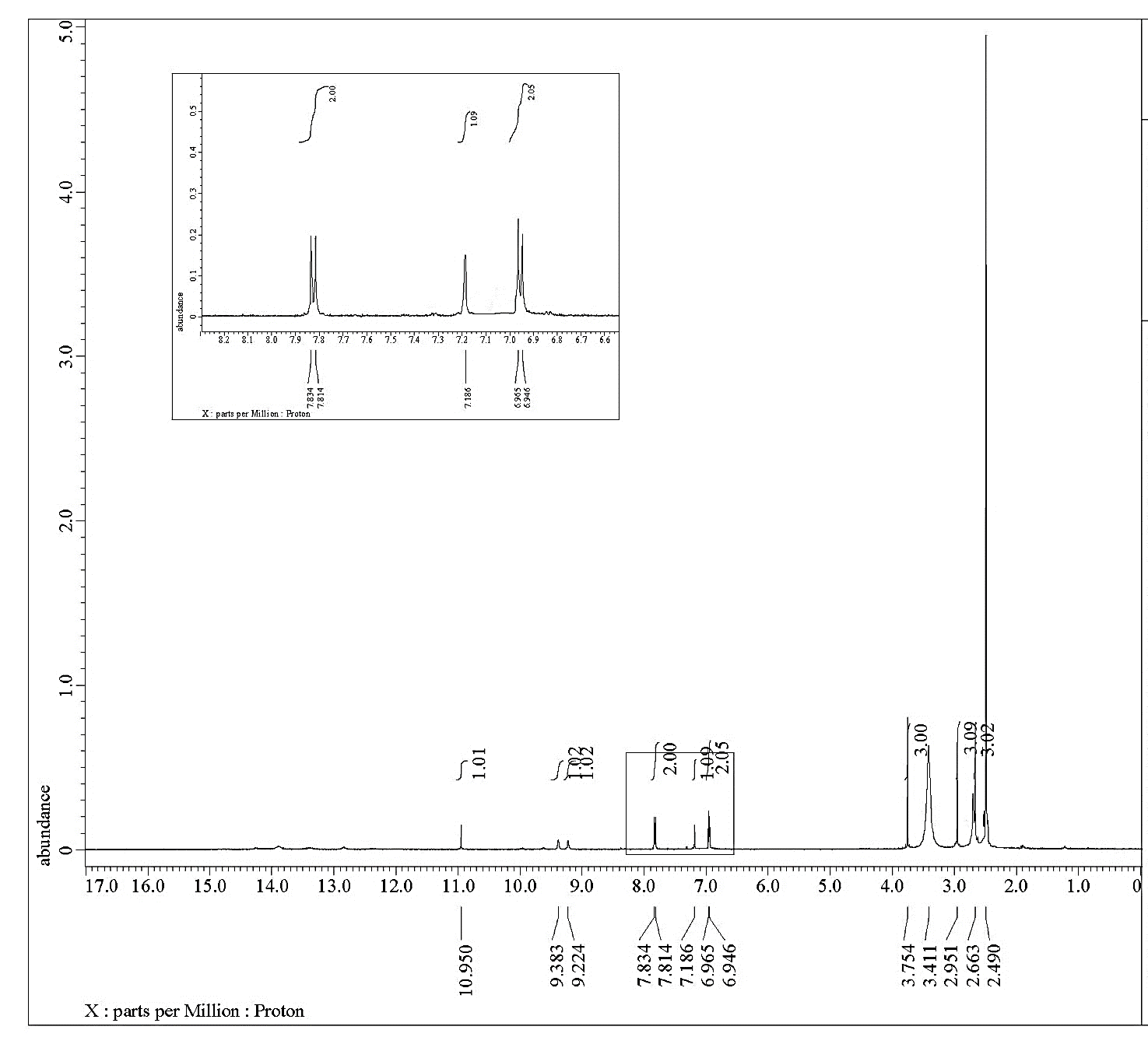
**

**Figure (S11): ^1^H NMR spectrum of pyridopyrazolo-triazine compound (3d)**

**
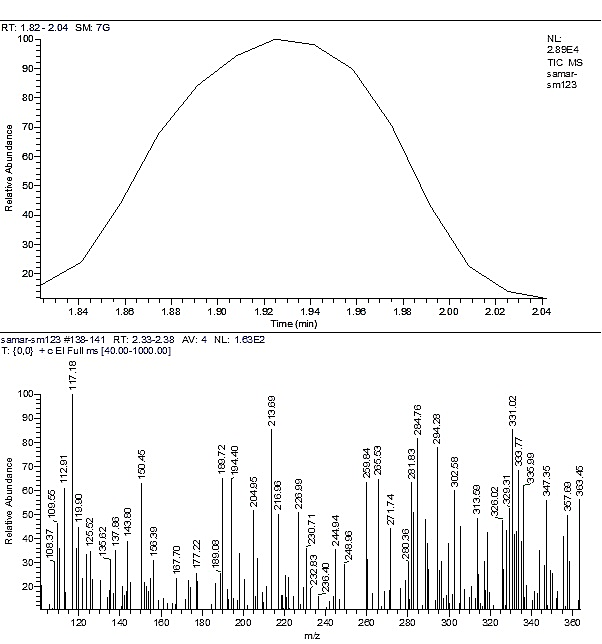
**

**Figure (S12): Mass spectrum of pyridopyrazolo-triazine compound (3d)**

**
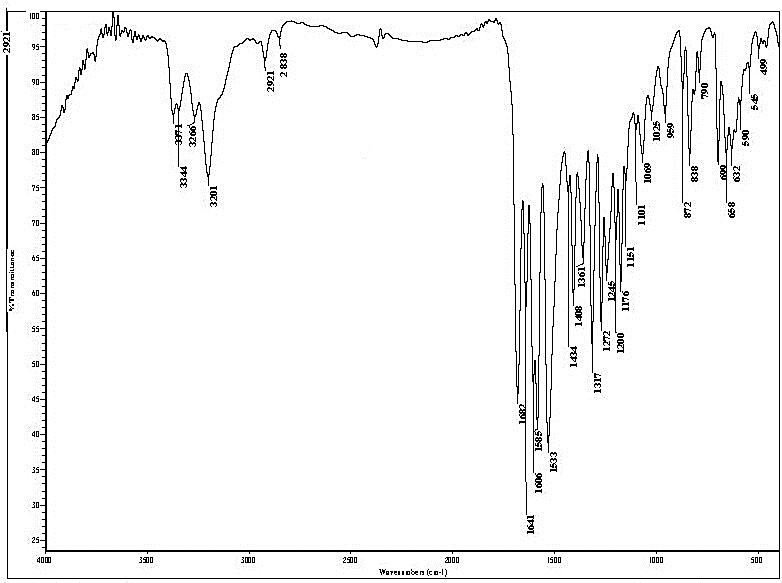
**

**Figure (S13): IR spectrum of pyridopyrazolo-triazine compound (3e)**

**
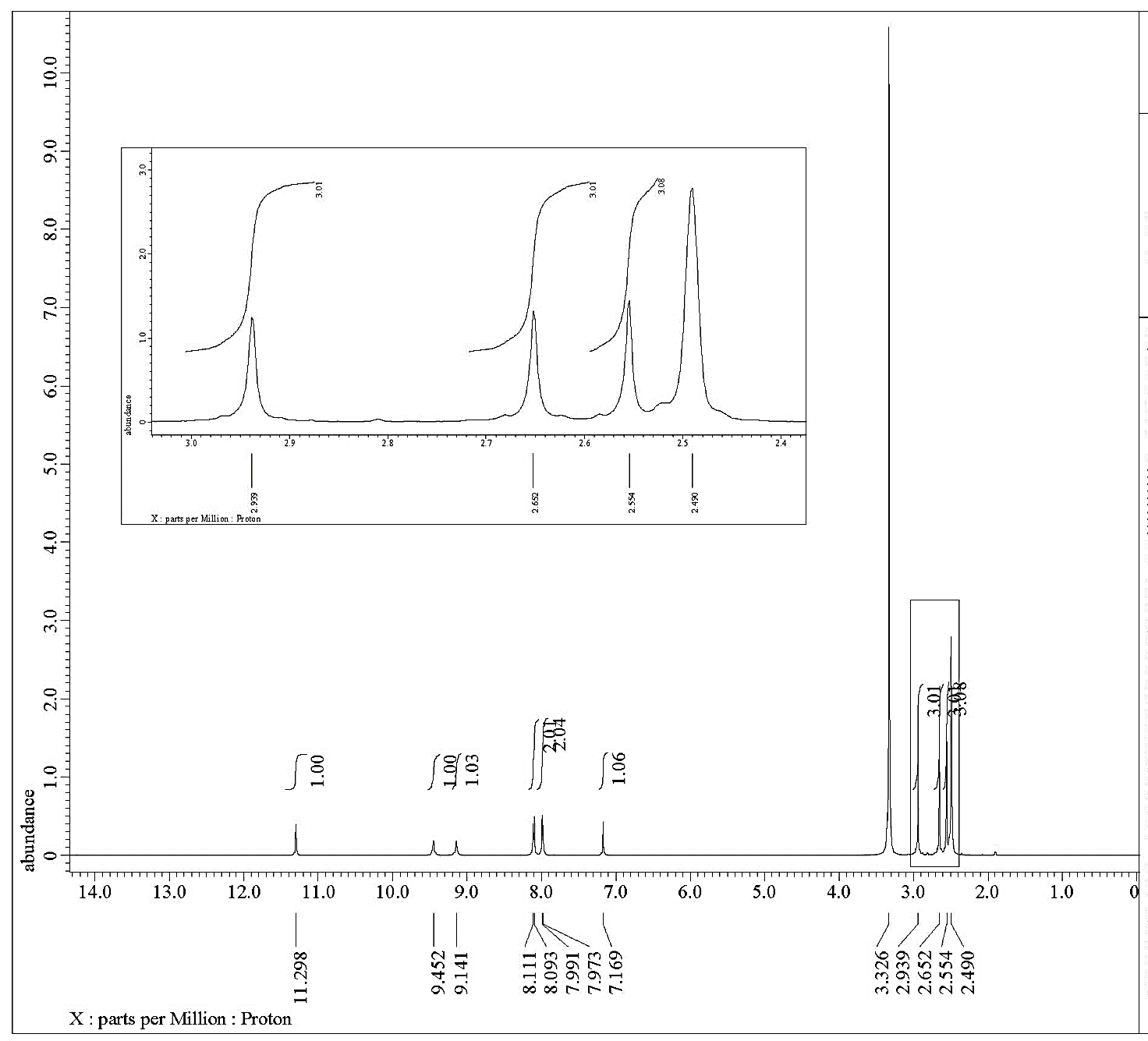
**

**Figure (S14): ^1^H NMR spectrum of pyridopyrazolo-triazine compound (3e)**

**Figure (S15): Mass spectrum of pyridopyrazolo-triazine compound (3e)**

**
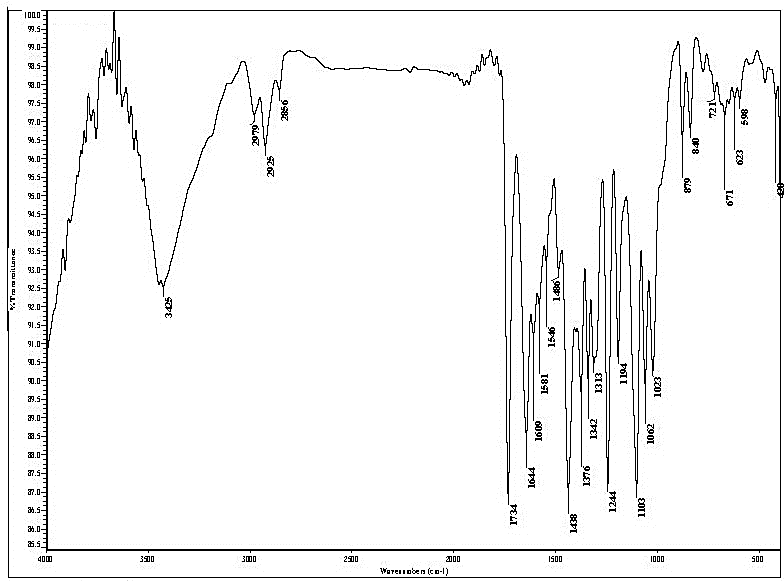
**

**Figure (S16): IR spectrum of pyridopyrazolo-triazine compound (5a)**

**
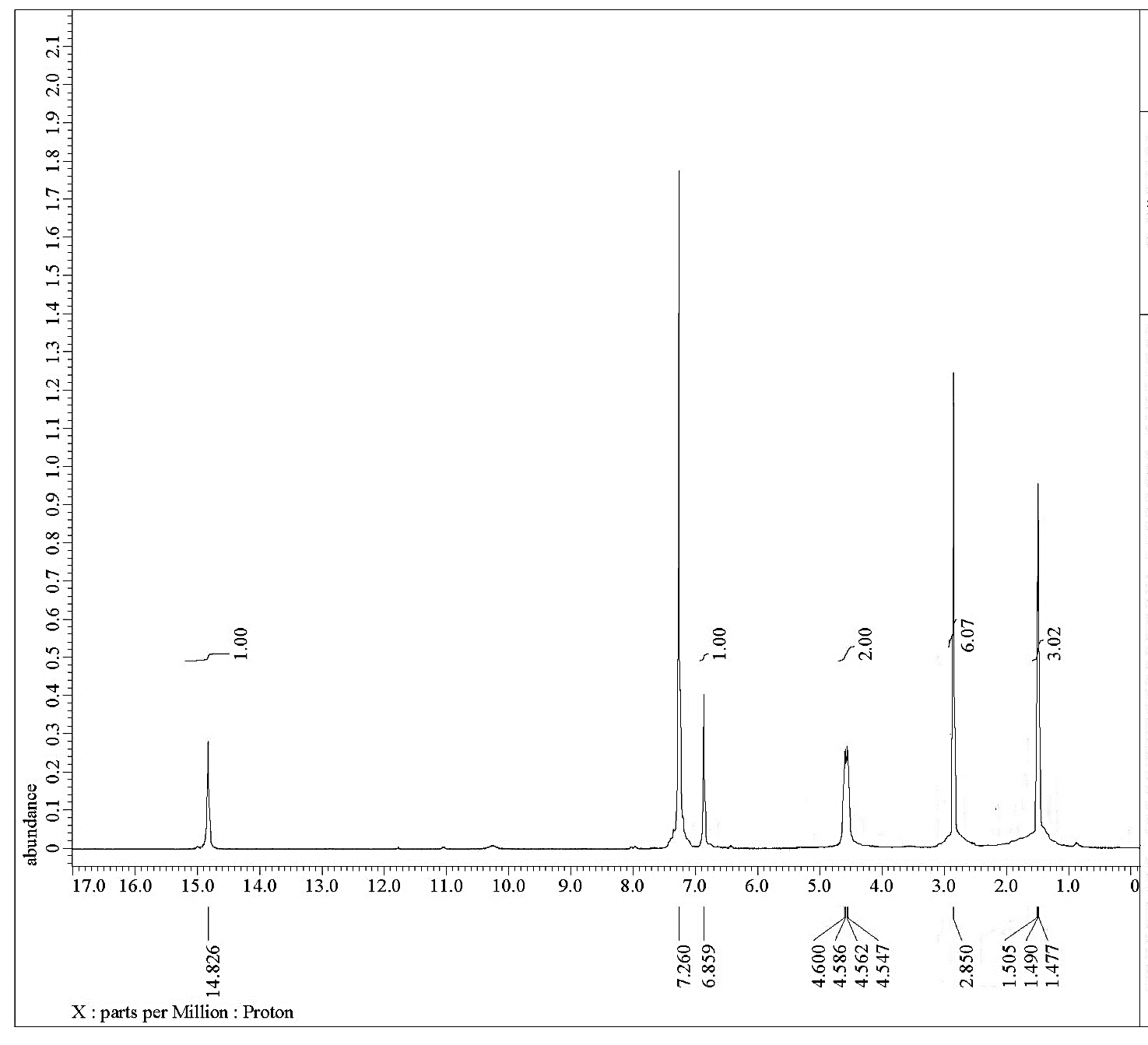
**

**Figure (S17): ^1^H NMR spectrum of pyridopyrazolo-triazine compound (5a)**

**
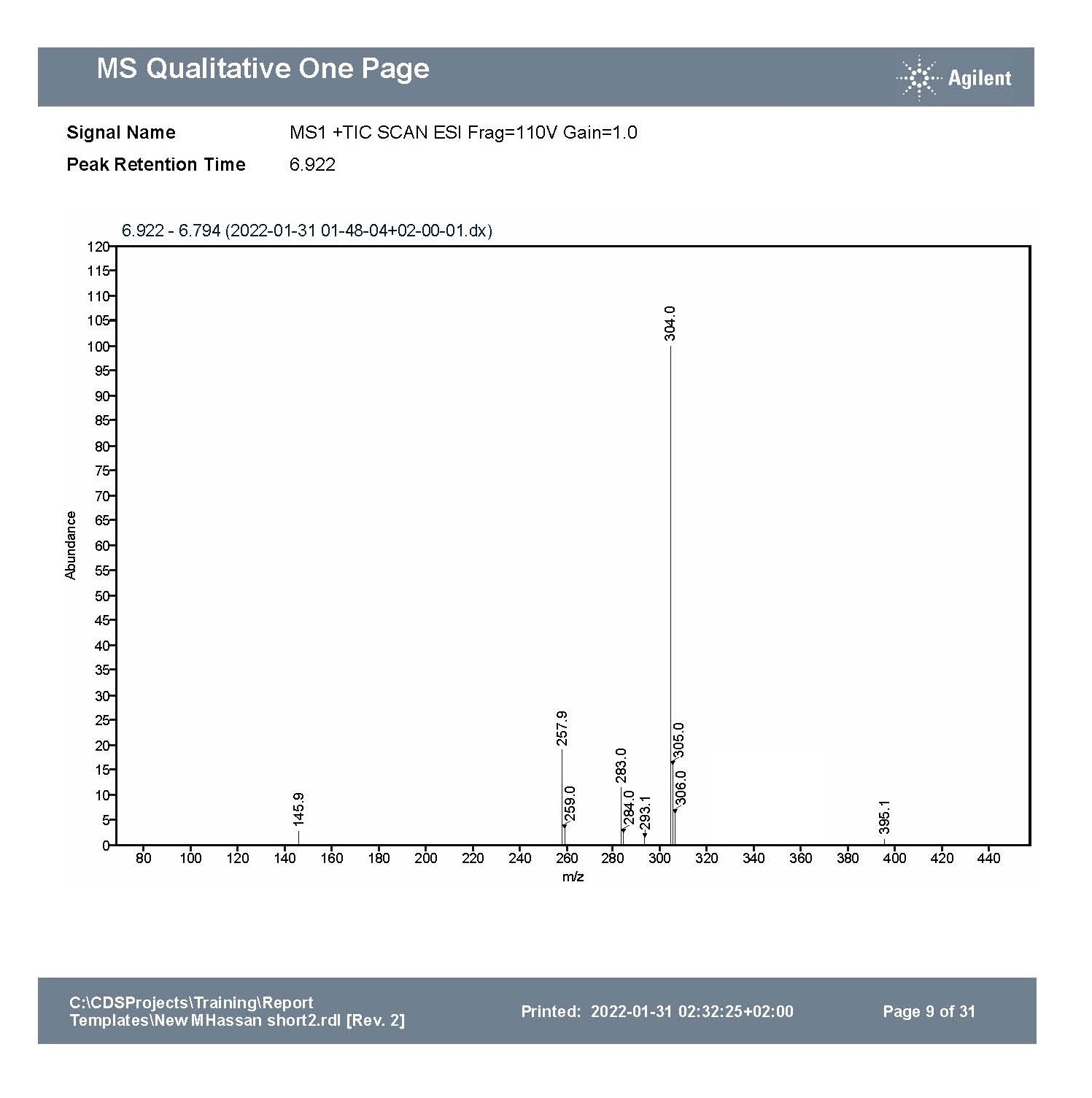
**

**Figure (S18): Mass spectrum of pyridopyrazolo-triazine compound (5a)**

**
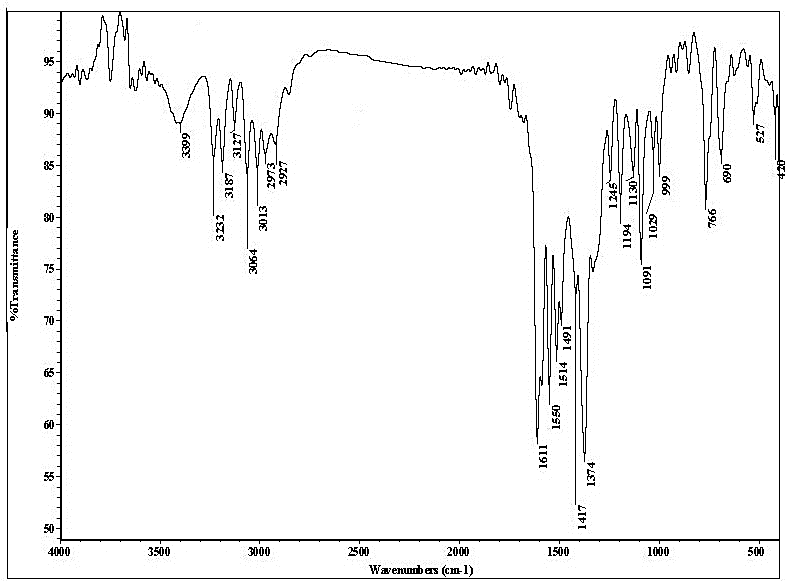
**

**Figure (S19): IR spectrum of pyridopyrazolo-triazine compound (6a)**

**
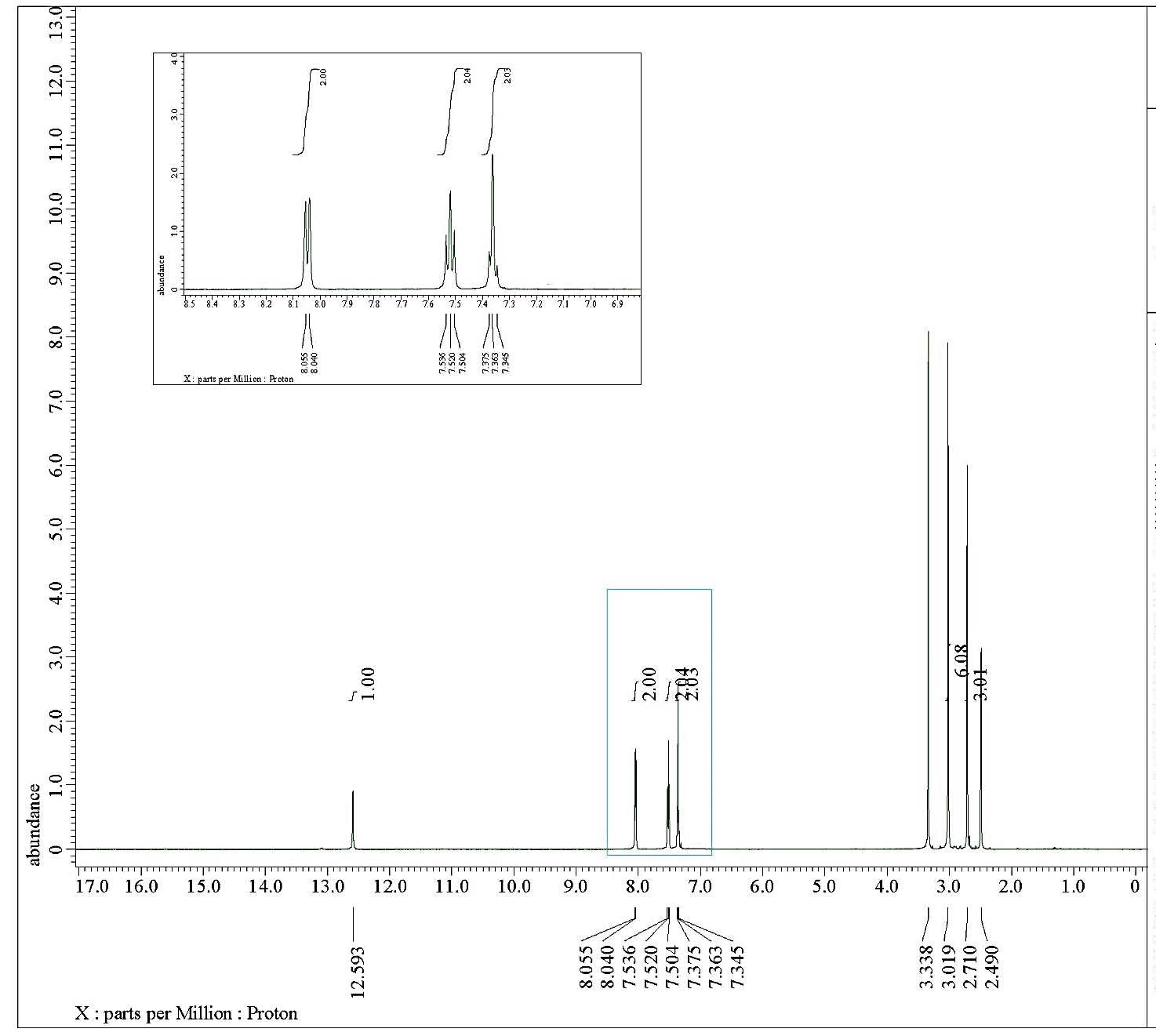
**

**Figure (S20): ^1^H NMR spectrum of pyridopyrazolo-triazine compound (6a)**

**Figure (S21): Mass spectrum of pyridopyrazolo-triazine compound (6a)**

**
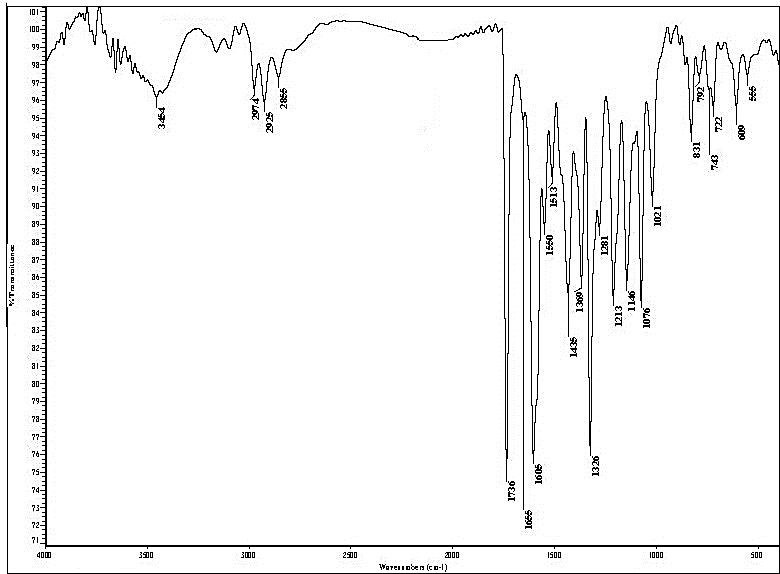
**

**Figure (S22): IR spectrum of pyridopyrazolo-triazine compound (7)**

**
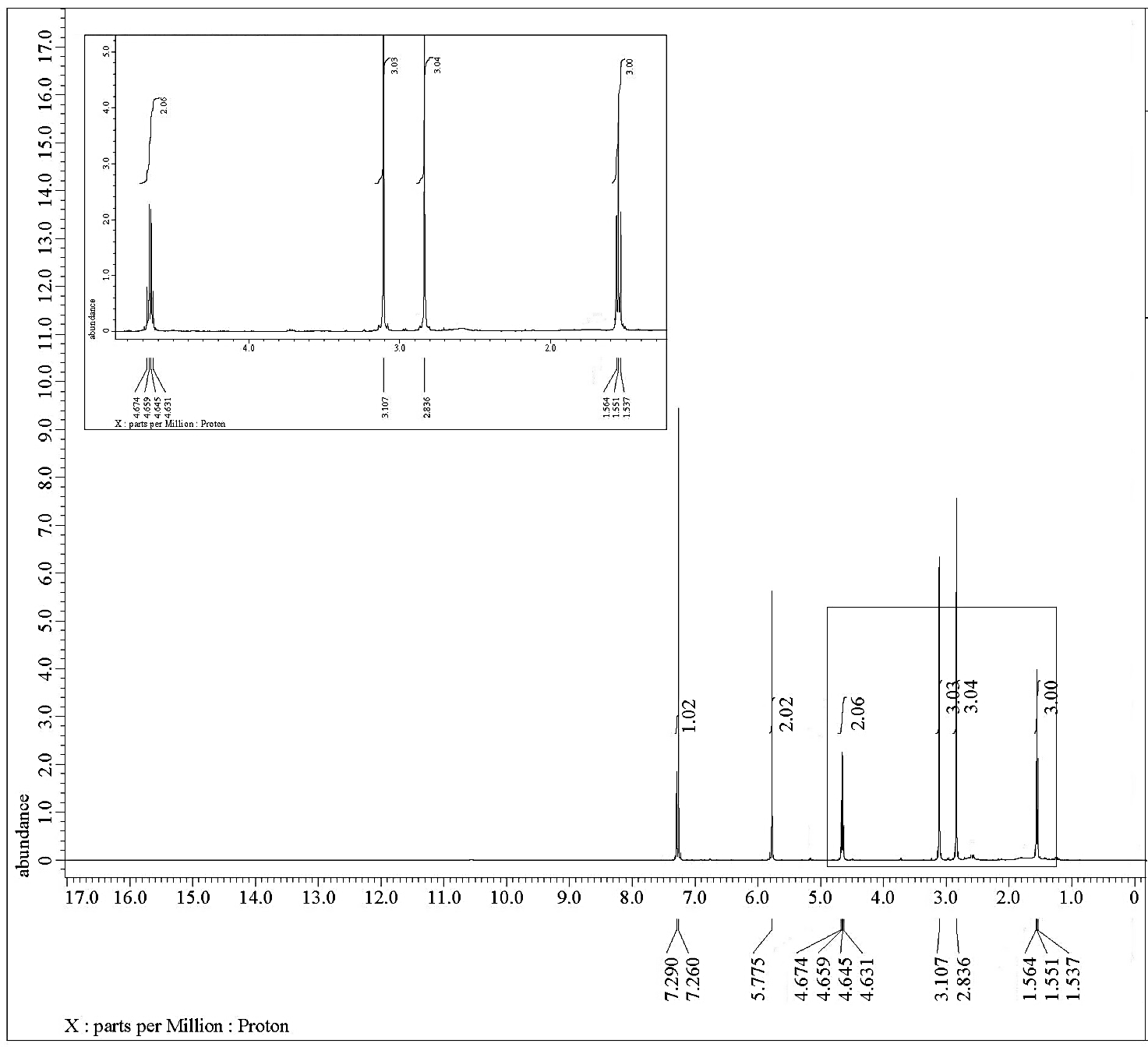
**

**Figure (S23): ^1^H NMR spectrum of pyridopyrazolo-triazine compound (7)**

**Figure (S24): Mass spectrum of pyridopyrazolo-triazine compound (7)**

**
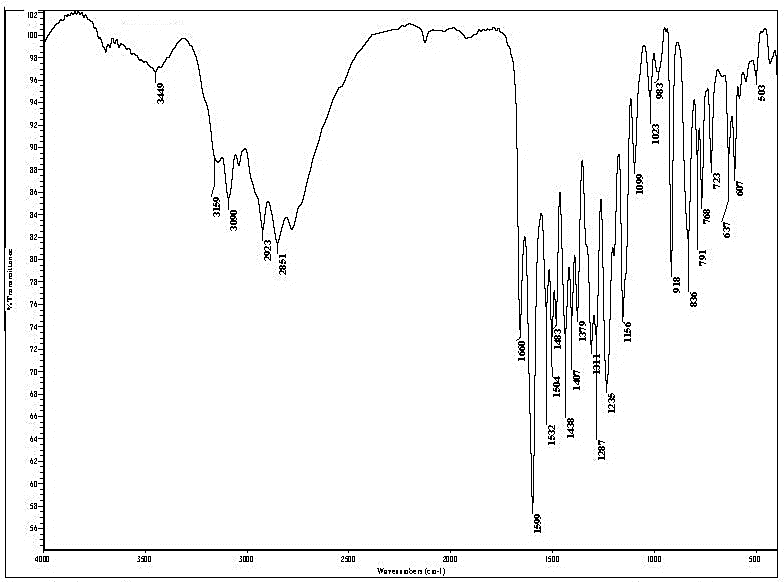
**

**Figure (S25): IR spectrum of pyridopyrazolo-triazole compound (9a)**

**
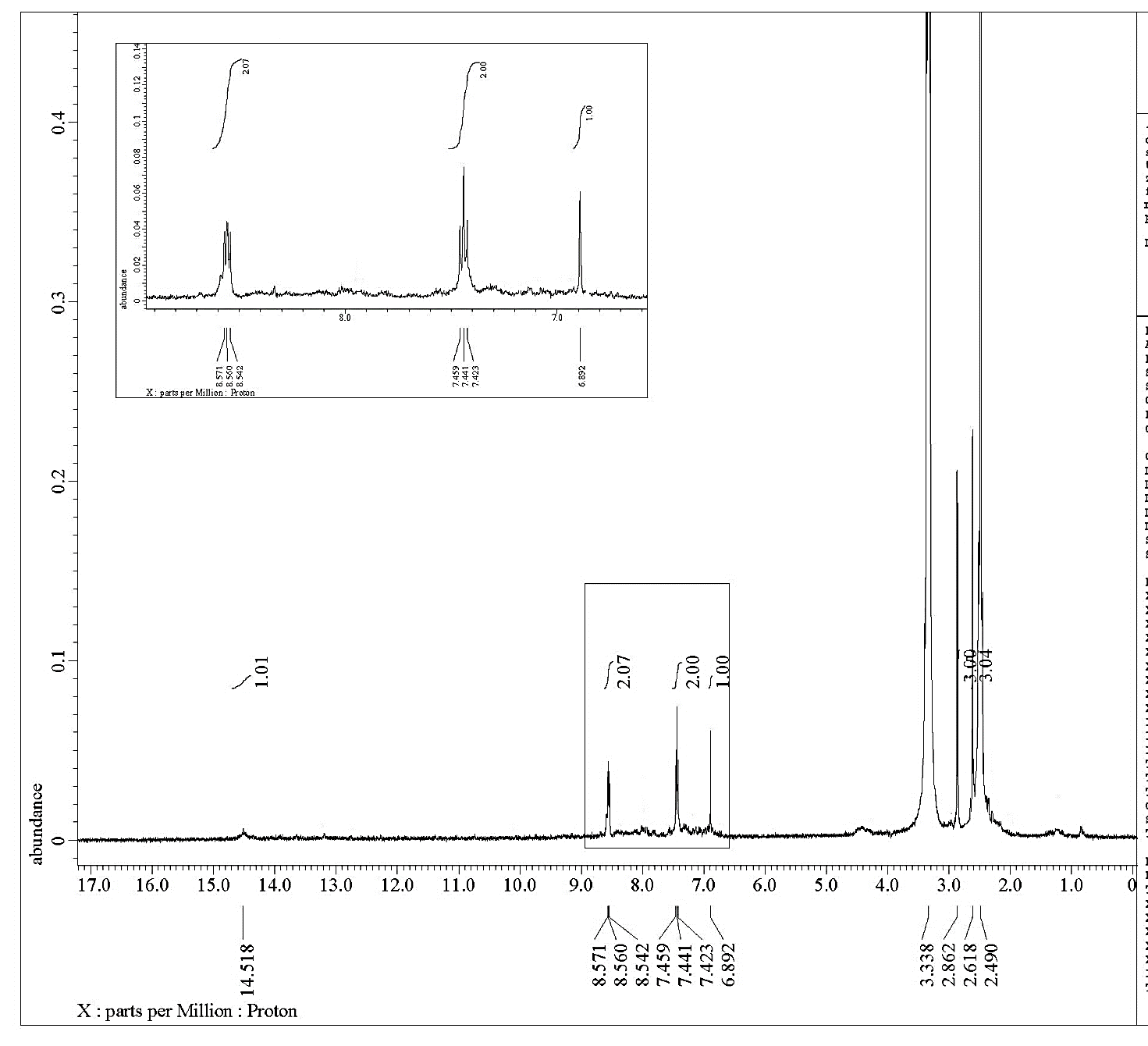
**

**Figure (S26): ^1^H NMR spectrum of pyridopyrazolo-triazole compound (9a)**

**Figure (S27): Mass spectrum of pyridopyrazolo-triazole compound (9a)**

**
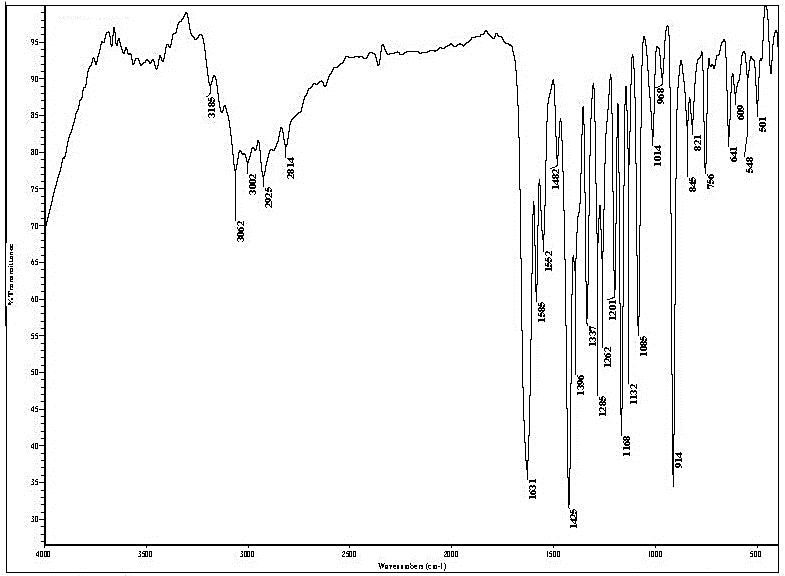
**

**Figure (S28): IR spectrum of pyridopyrazolo-triazole compound (9b)**

**
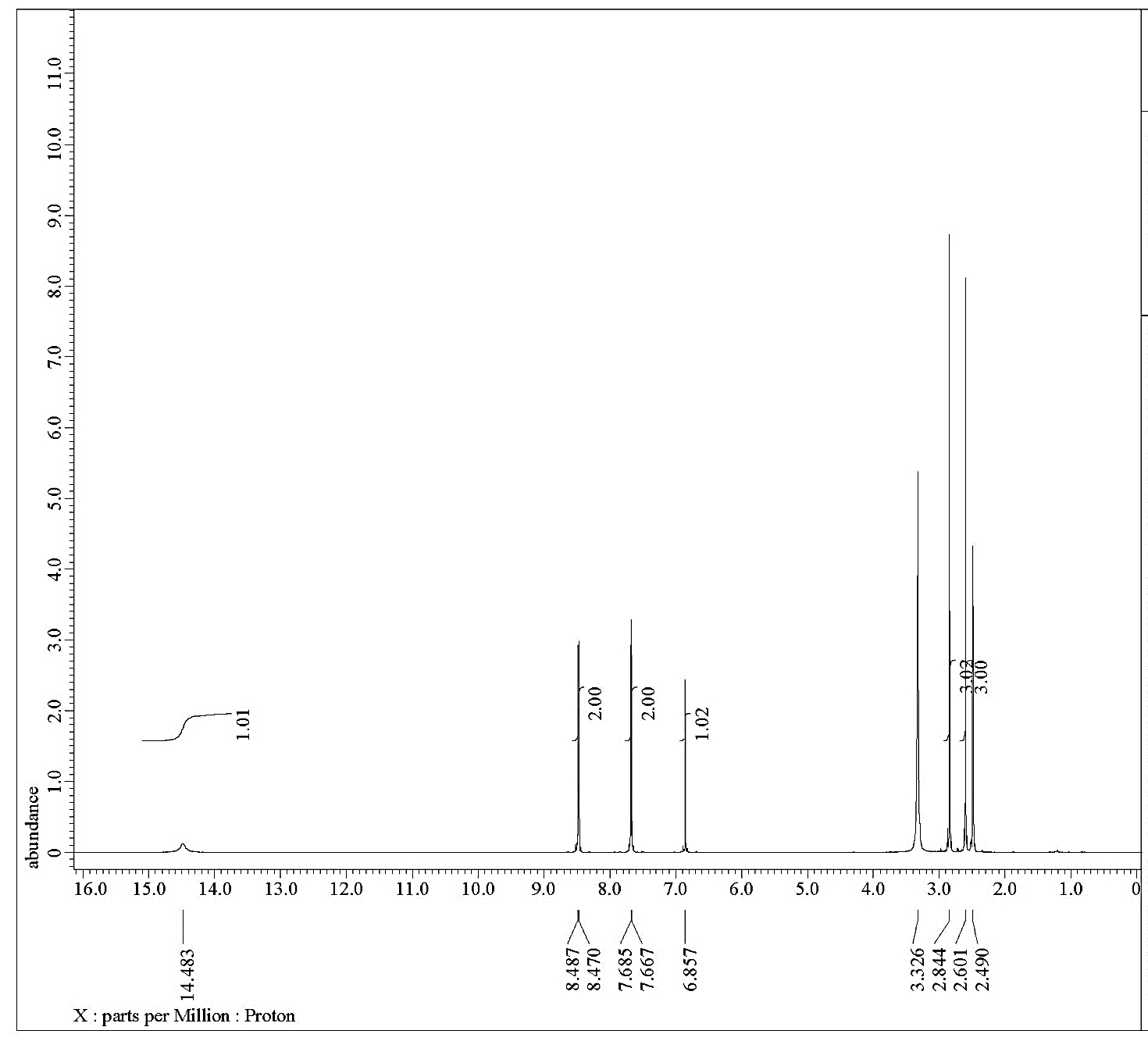
**

**Figure (S29): ^1^H NMR spectrum of pyridopyrazolo-triazole compound 9b**

**Figure (S30): Mass spectrum of pyridopyrazolo-triazine compound (9b)**

**
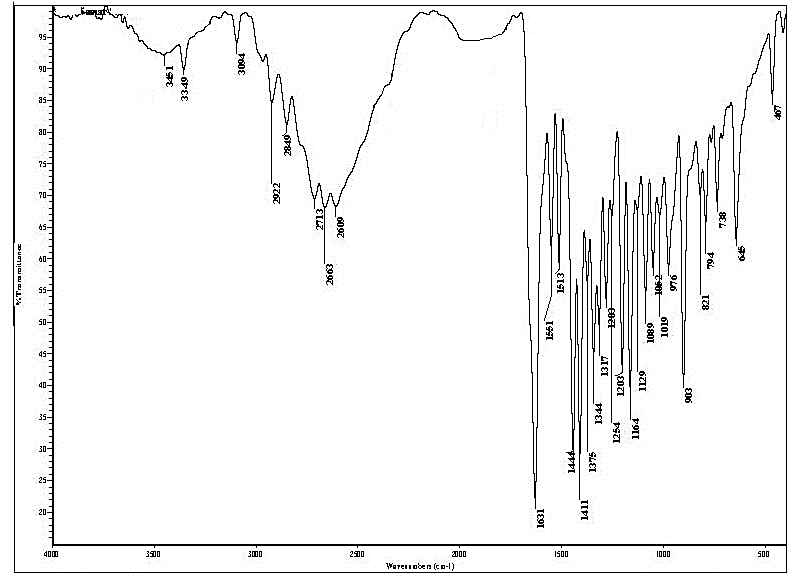
**

**Figure (S31): IR spectrum of pyridopyrazolo-triazine compound (10)**

**
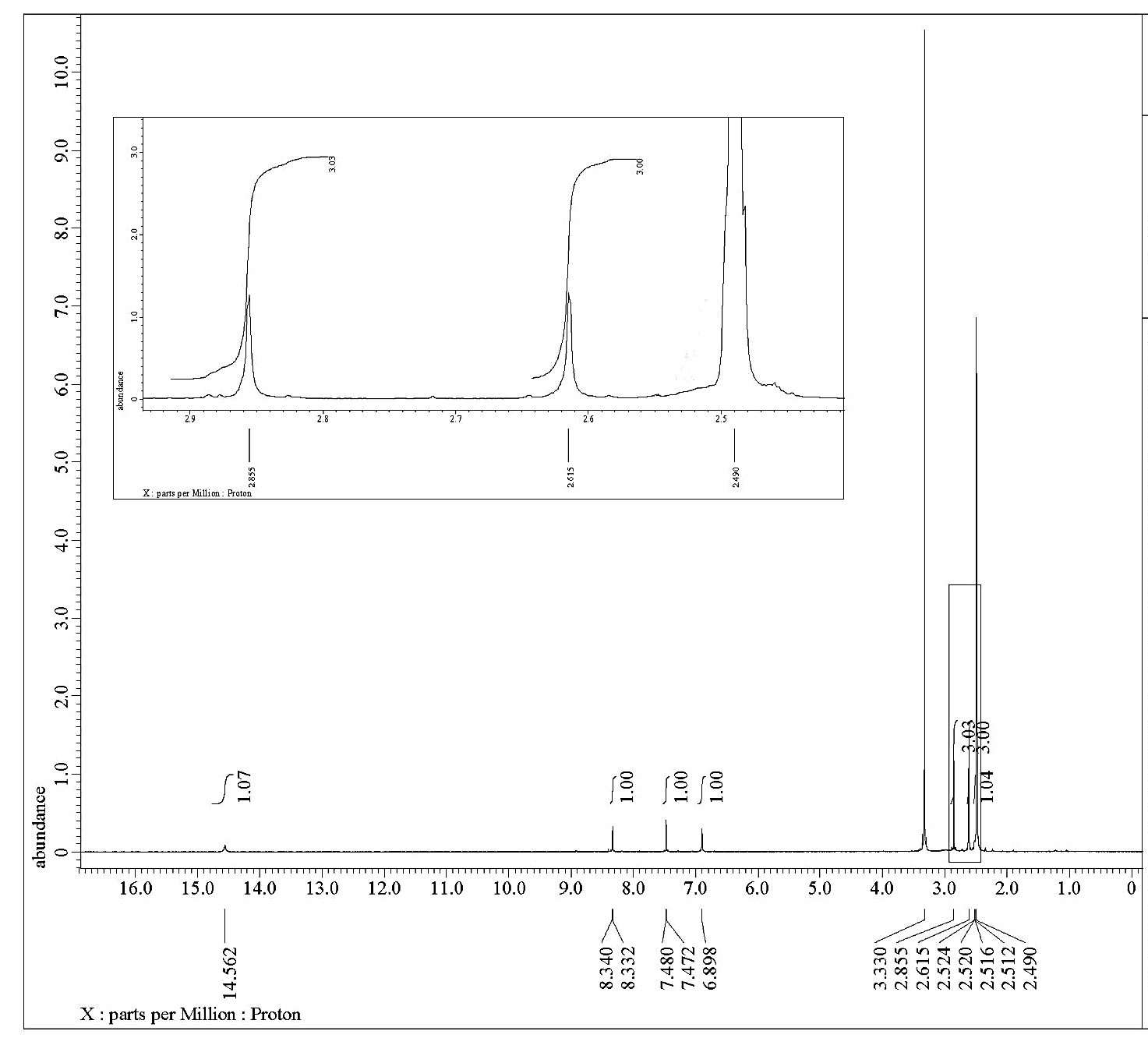
**

**Figure (S32): ^1^H NMR spectrum of pyridopyrazolo-triazine compound (10)**

**Figure (S33): Mass spectrum of pyridopyrazolo-triazine compound (10)**

**
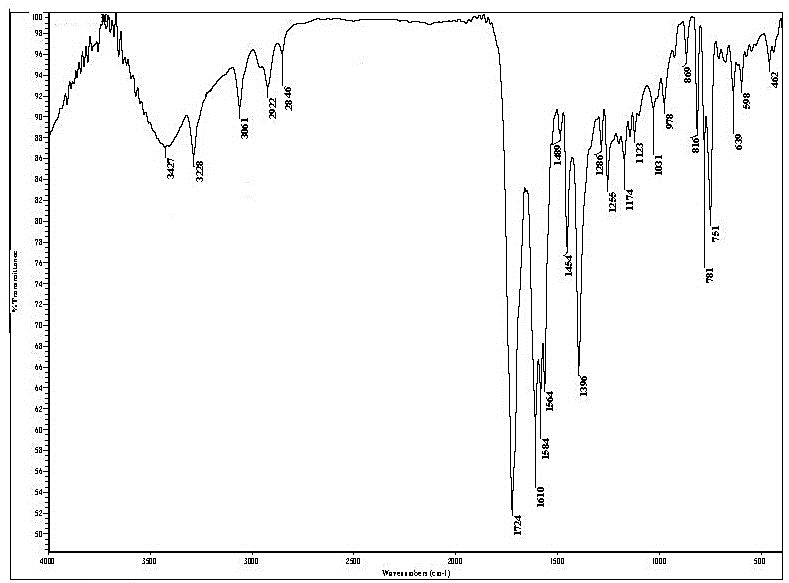
**

**Figure (S34): IR spectrum of pyridopyrazolo-triazine compound (11)**

**
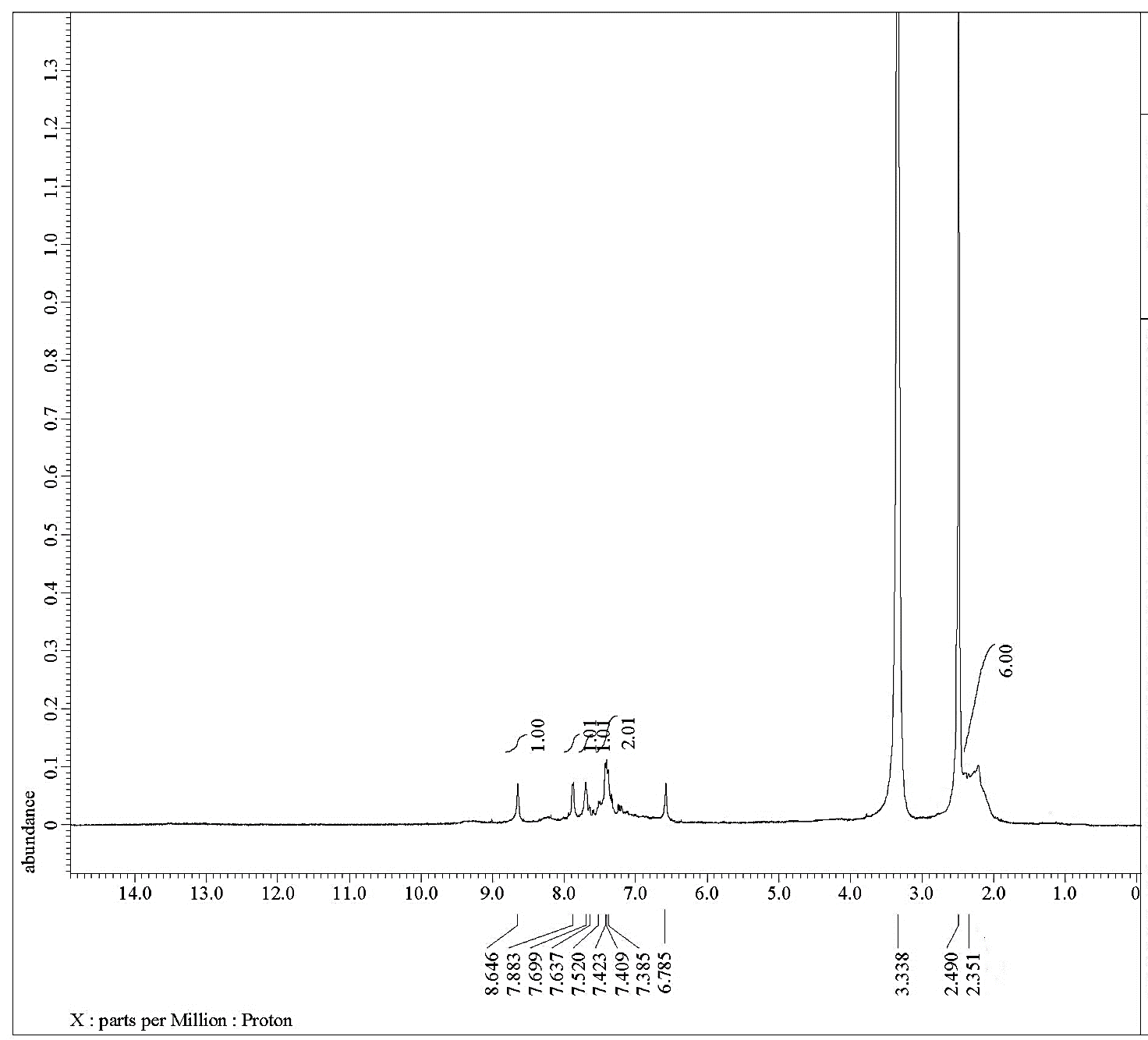
**

**Figure (S35): ^1^HNMR spectrum of pyridopyrazolo-triazine compound (11)**

**Figure (S36): Mass spectrum of pyridopyrazolo-triazine compound (11)**

**
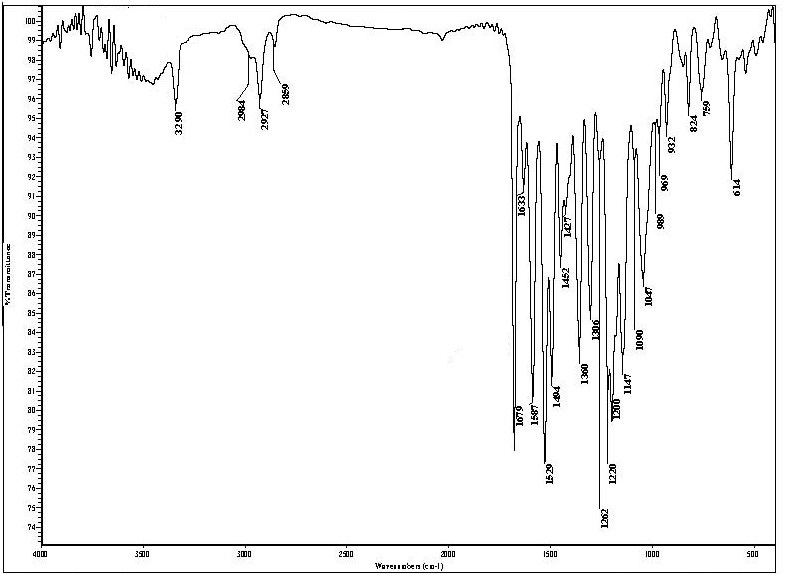
**

**Figure (S37): IR spectrum of pyridopyrazolo-triazine compound (12)**

**
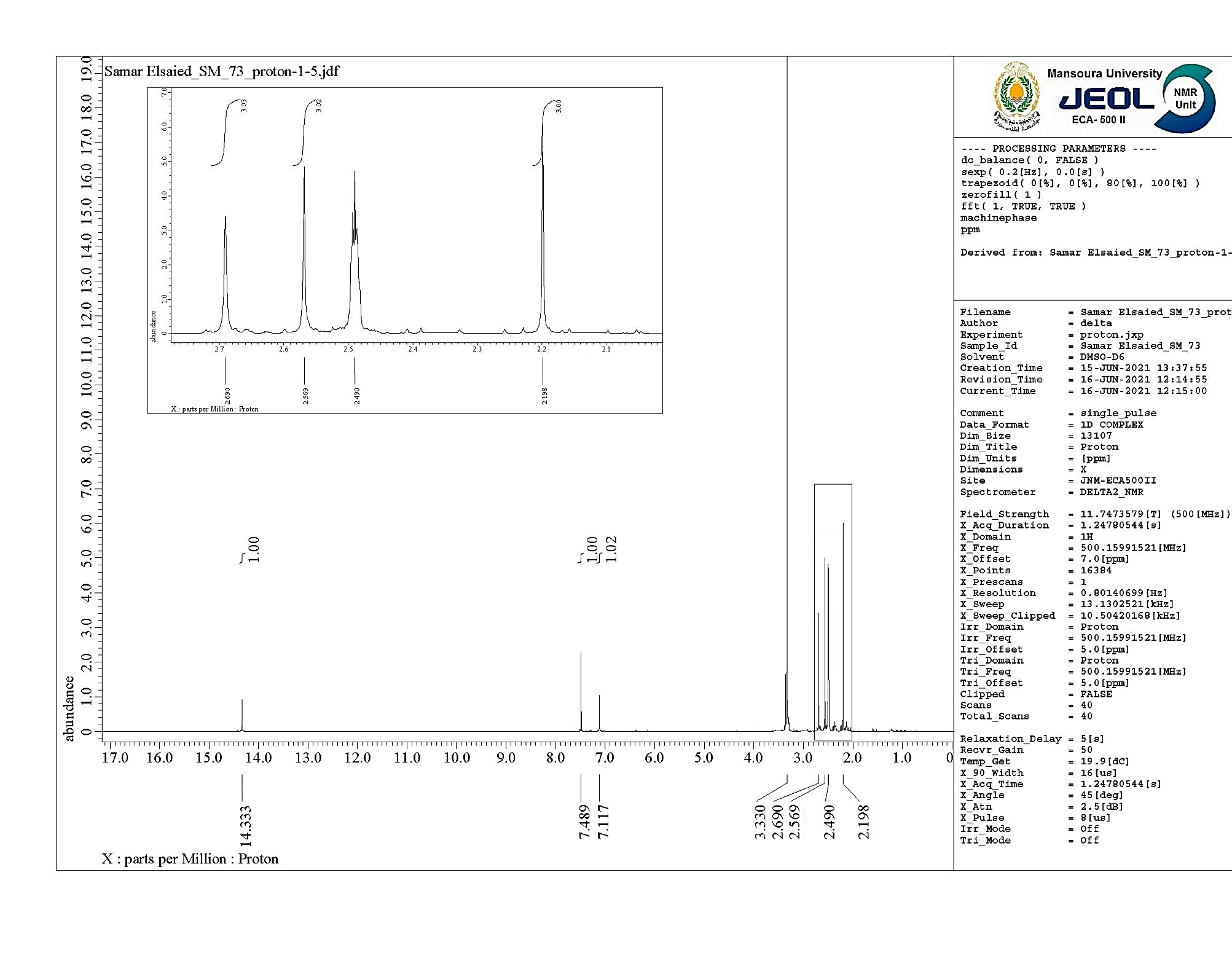
**

**Figure (S38): ^1^H NMR spectrum of pyridopyrazolo-triazine compound (12)**

**
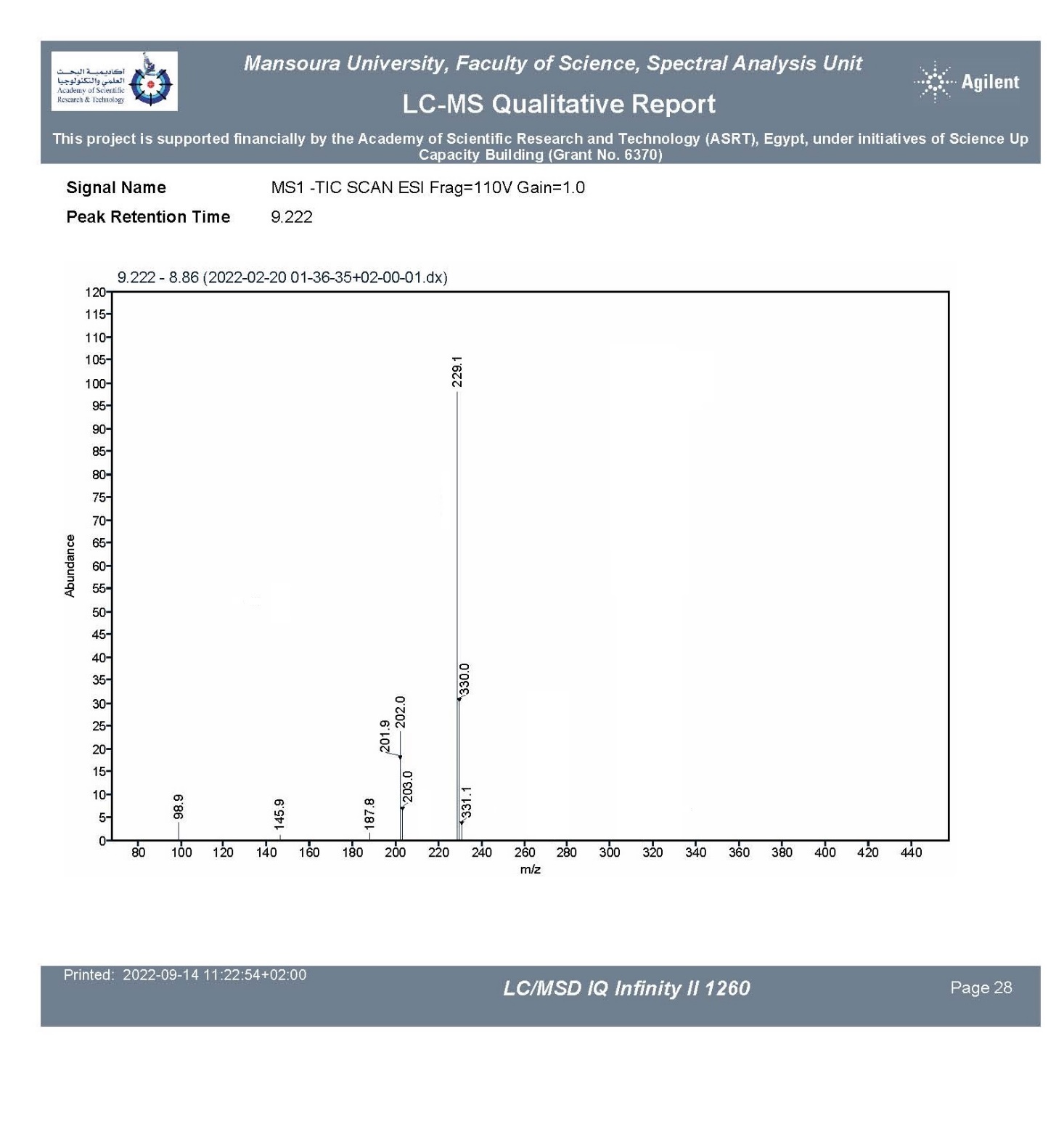
**

**Figure (S39): Mass spectrum of pyridopyrazolo-triazine compound (12)**

**2. Docking analysis**

| 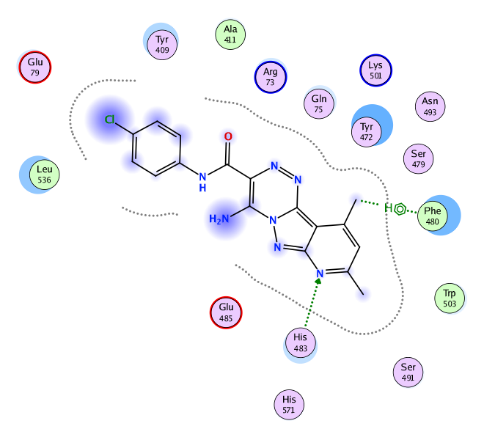 | 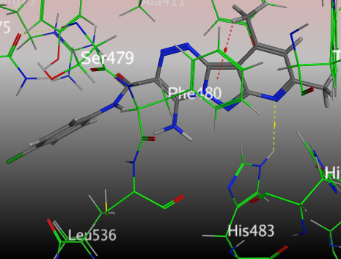 |
| --- | --- |
| 2D | 3D |

**Figure (S40):** The binding interactions of compound **3b** with active sites of (PDB ID: 5IVE).

| 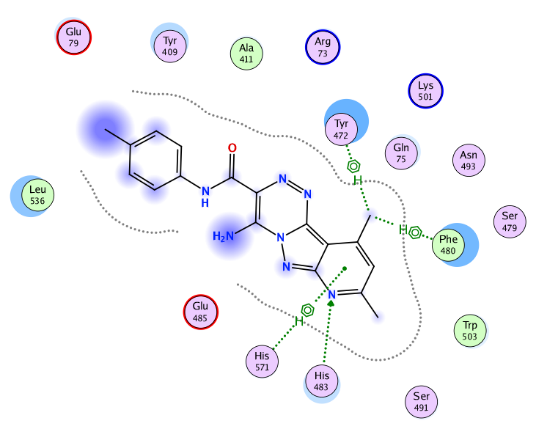 | 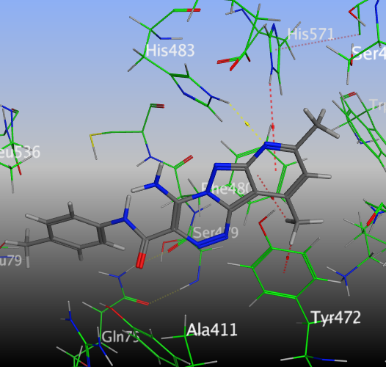 |
| --- | --- |
| 2D | 3D |

**Figure (S41):** The binding interactions of compound **3c** with active sites of (PDB ID: 5IVE).

| 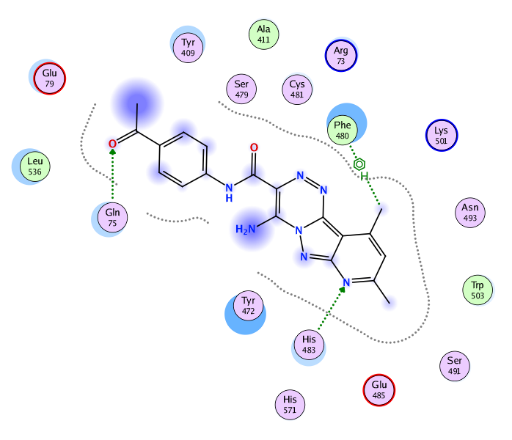 | 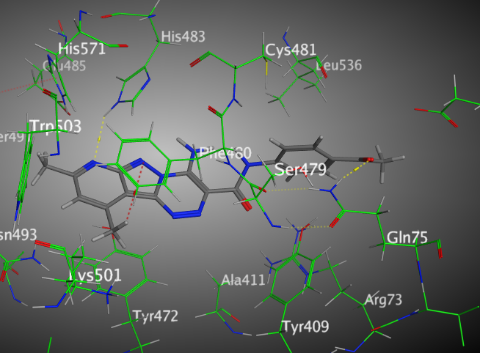 |
| --- | --- |
| 2D | 3D |

**Figure (S42):** The binding interactions of compound **3e** with active sites of (PDB ID: 5IVE).

| 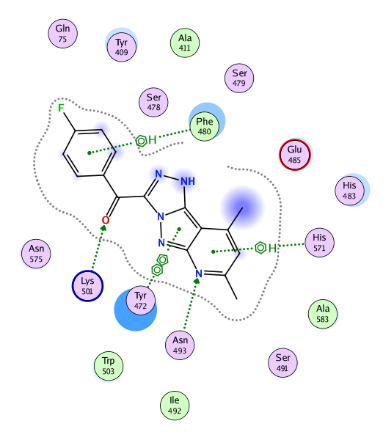 | 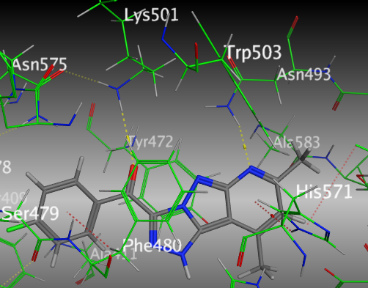 |
| --- | --- |
| 2D | 3D |

**Figure (S43):** The binding interactions of compound **9a** with active sites of (PDB ID: 5IVE).

| 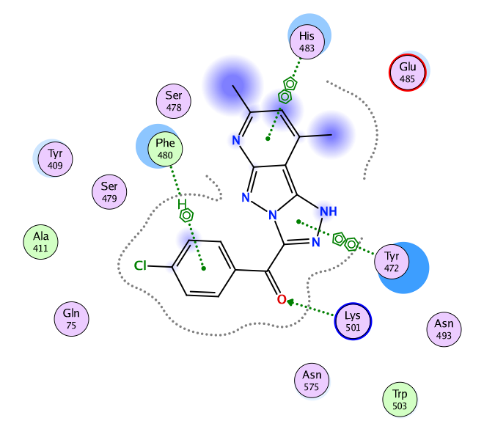 | 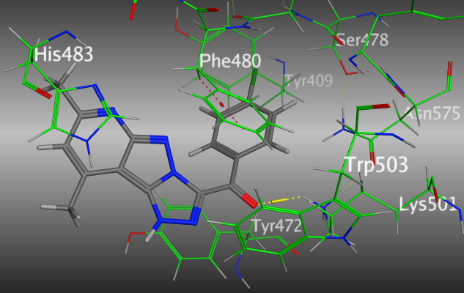 |
| --- | --- |
| 2D | 3D |

**Figure (S44):** The binding interactions of compound **9b** with active sites of (PDB ID: 5IVE).

| 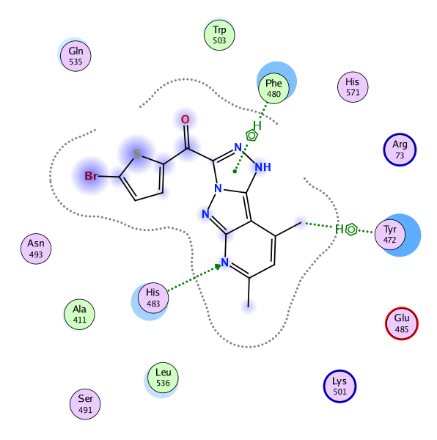 | 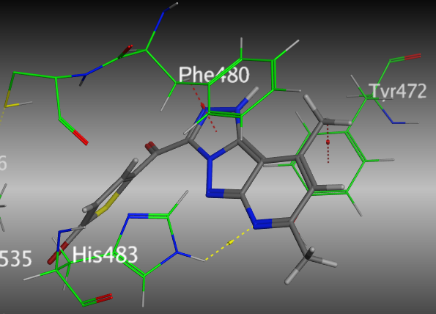 |
| --- | --- |
| 2D | 3D |

**Figure (S45):** The binding interactions of compound **10** with active sites of (PDB ID: 5IVE).

| 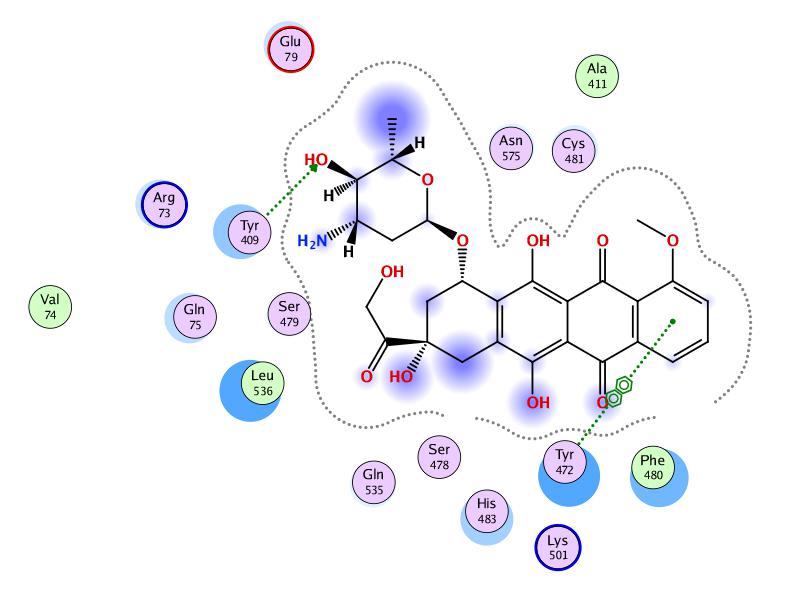 | 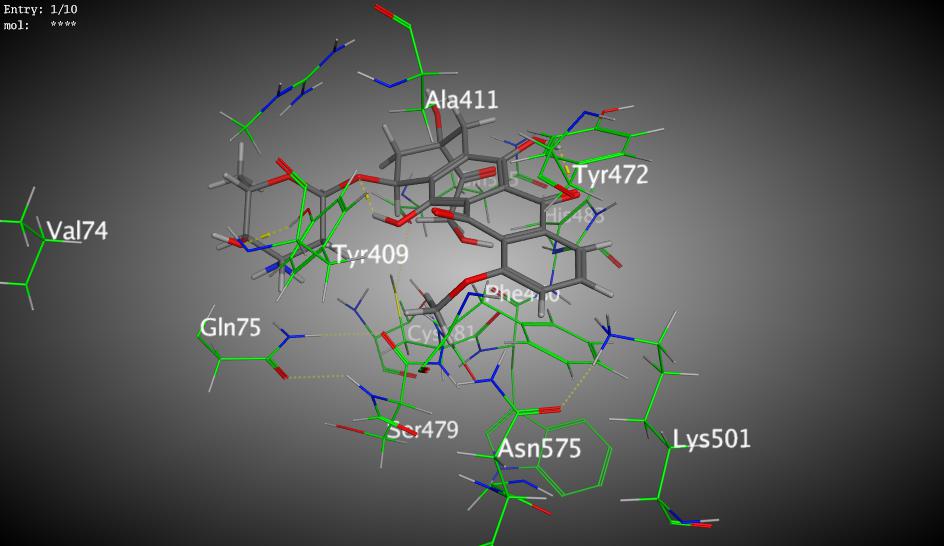 |
| --- | --- |
| 2D | 3D |

**Figure (S46):** The binding interactions of doxorubicin with active sites of (PDB ID: 5IVE).
